# Optical Correlators for Cryptosystems and Image Recognition: A Review

**DOI:** 10.3390/s23020907

**Published:** 2023-01-12

**Authors:** Andrei Drăgulinescu

**Affiliations:** Electronic Technology and Reliability Department, University Politehnica of Bucharest, 060042 Bucharest, Romania; andrei.dragulinescu@upb.ro

**Keywords:** optical correlator, joint transform correlator, VanderLugt correlator, Fourier transform, encryption, cryptosystem, image recognition, face recognition

## Abstract

Optical correlators are efficient optical systems that have gained a wide range of applications both in image recognition and encryption, due to their special properties that benefit from the optoelectronic setup instead of an all-electronic one. This paper presents, to the best of our knowledge, the most extensive review of optical correlators to date. The main types are overviewed, together with their most frequent applications in the newest contributions, ranging from security uses in cryptosystems, to medical and space applications, femtosecond pulse detection and various other image recognition proposals. The paper also includes a comparison between various optical correlators developed recently, highlighting their advantages and weaknesses, to gain a better perspective towards finding the best solutions in any specific domain where these devices might prove highly efficient and useful.

## 1. Introduction

### 1.1. Background and Motivation

Optical correlators are optical systems that accomplish an operation called optical correlation, which can be defined as a process that involves the following steps: first, a multiplication is performed between the image of the target and a reference image, included in a database; secondly, a comparison is carried out between the peak-to-correlation energy (PCE) and a threshold, previously set. A decision will finally be taken, based on this comparison that determines the correspondence between the two images (target and reference). Therefore, there are two steps, the first one performed in the spectral domain, whereas the second one is in the spatial domain. Finally, the third step involves the decision, the most frequent criterion for decision being the PCE. If the similarity between the images overcomes the threshold, the correlation result will present a bright spot, named peak [1,2,3]. They are used for applications which vary from image correlation calculus to various applications of data analysis [4], such as analysis of DNA (deoxyribonucleic acid).

Mainly two applications directions have differentiated as concerns the optical correlation for images. The first one involves the design of novel correlation filters to improve the methods of correlation, whereas the second deals with biometric applications, in turn with several main directions: face recognition (the most frequent one, with early and recent applications in building access control [5], video surveillance, unmanned vehicles, identification of law breakers etc. [1]), recognition of moving targets (e.g., boats, aircrafts, missiles) in a complex background, detection of fingerprints, robotics, military (defense) uses, medical applications for an automatic early detection of various diseases, by detection and matching of various biological samples [1,6,7,8,9,10].

The methods that employ optical correlation have powerful competitors: numerical methods (neural networks, SVM—support vector machine etc.) [6]. However, optical correlation methods have retained their attractiveness and wide use due to their good performance and their ease of use, since correlation is based only on few mathematical operations (generally, two Fourier transforms and, in the frequency domain, one multiplication) [6]. As compared to optical correlation, numerical methods based on Deep Learning (DL), while also performing well, have a significantly more complex algorithm, the learning processes take a much longer time, the implementation is more difficult and requires an increased number of computational resources [6]. However, deep learning techniques have also been more recently proposed in an integration with optical methods, benefiting from the advantages of both [11]. Deep learning methods can address the problem of information loss in classical techniques of ciphertext compression [12]. A deep convolutional network was successfully used for repairing the compression artifact appearing in the image [13]. DL techniques achieved a significant improvement of the images transmitted with ghost-imaging methods [14]. DL was also employed for the reconstruction of the ciphertext image that had been previously compressed into a 1D encrypted signal, using however a relatively complicated optical setup [15]. More recently, a deep neural network (DNN) was used in a novel setup, where the compression of the ciphertext was performed by a collaboration between the JPEG2000 standard and the bilinear interpolation method, whereas the decompression of the ciphertext was performed by the DNN, with results significantly better than state-of-art standards of compression, such as JPEG2000 and JPEG [12].

As concerns the systems that perform optical correlation, one can mention that among their advantages are their very good capabilities of data transfer speed and enhanced capacity for analysis of data [4]. By using optics instead of numerical solutions, one can benefit from the possibility of using its intrinsic parallelism of processing, from a wide range of specific optical parameters that can be controlled (quantum properties, wavelength, phase, polarization), from noise robustness, increased discrimination ability, shift invariance and less strict requirements of power consumption than in an electronic computer, where electrical processing and cooling require a high amount of energy consumption [9,16,17,18,19,20]. As concerns the methods of optical correlation for image recognition, the implementation is easy, both numerically (by two Fourier transforms) and optically (with the use of two lenses) [6]. Moreover, as compared to other methods of pattern recognition, computer-based, optical correlation has a higher speed of operation, improved stability, a better resistance to electromagnetic interference and a higher bandwidth [7].

However, as concerns the disadvantages, the main ones in optical image recognition are the possibility of taking wrong decisions when analyzing the shape, location and height of the peaks of correlation, and the high sensitivity of correlation when the images are rotated or scaled [6]. Other drawbacks are the very strict requirements concerning the alignment of the setup components, effects of aberrations, the cost of some of the optoelectronic devices used, such as the spatial light modulator (SLM) etc. [1]. Another problem that may constitute an obstacle in the implementation of such techniques in optoelectronics is posed by the redundancy of the transformations, because optical correlation involves two transformations: one from the spatial to the spectral domain, followed by another one from the spectral to the spatial domain [1]. Moreover, generally optical components present a slow response and are bulky. To surpass these last two drawbacks, components based on metamaterials have been proposed, with a faster response and smaller dimensions [16,17].

### 1.2. Objectives of the Review

The review has the following objectives:

- To thoroughly present the theory and types of the optical correlators.
- To present the recent implementations of optical correlators in domains such as:
  - cryptosystems and security applications;
  - image recognition;
  - object recognition;
  - target recognition.
- To present the open challenges associated with the implementation of optical correlators.

### 1.3. Paper Organisation

The rest of this paper is organized as follows: In Section 2, I present the theory, sub-types, and main applications for the main two types of optical correlators (JTC and VLC) and for other less frequently used correlator types. Section 3 includes recent implementations of these types of optical correlators in security and image recognition applications and in several other domains (medicine, astronomy, sports etc.). The challenges and the open issues are discussed in Section 4. Finally, Section 5 concludes the paper.

## 2. Classification and Overview of the Principles of Optical Correlators

There are several types of optical correlators, among which the most used are the joint transform correlator (JTC) and the VanderLugt correlator (VLC), also named the matched filter correlator (MFC) [3]. The VLC correlator was, historically, the first successful optical correlator, developed by VanderLugt in 1963. In this correlator type, the detection of a target in a scene can be performed automatically by using a filter where the complex conjugated Fourier transform of the object is performed. The images of the scene and a reference are compared, by means of the intensity of the correlation at the output. This device was also named the 4f-correlator, where f is the focal length of the two lenses of the setup, and 4f is the distance between the input and output planes [21]. The VLC correlator is still efficient in applications today; however, it has two important drawbacks: a very high sensitivity to the alignment of the components of the setup and the requirement of specialized hardware [3].

The JTC correlator was developed by Weaver and Goodman in 1966, to overcome the limitations of the VLC correlator [3]. In the JTC, both the scene and the reference are positioned in the input plane [21]. Although it is truly less sensitive to alignment issues, it is generally less compact, thus occupying more space than a similar VLC correlator [3]. An important advantage of the JTC over VLC is that it eliminates the need of the matched filter. On the other hand, the DC term (the zero-order one) is very large in the correlation output; thus, if it is not removed, it will lower the performance of the correlator. Two architectures that remove the zero-order term are the N0JTC (nonzero-order JTC) and the MZJTC (Mach–Zehnder JTC) [22].

**Figure 1:**
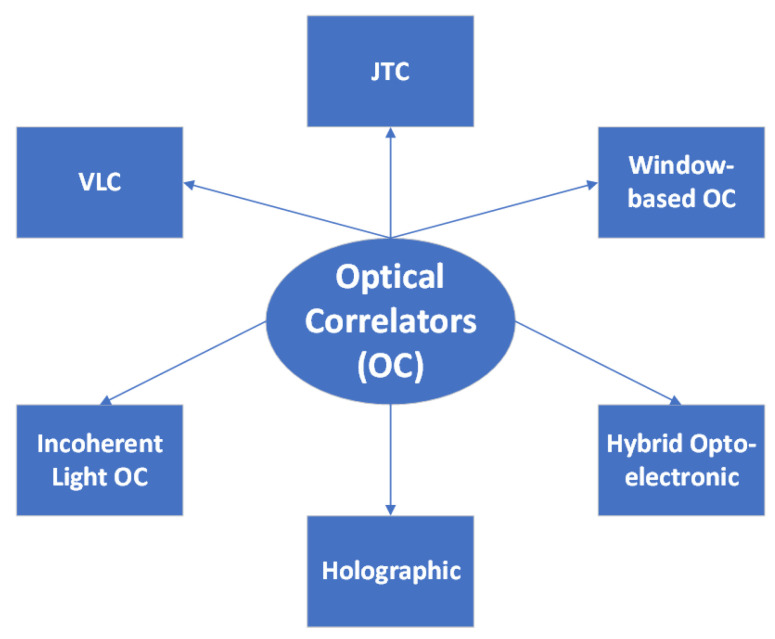
Optical correlator types.

This section consists of an analysis of the theory, types and most important applications of the JTC and VLC correlator, followed by other less frequently used correlator types: the optical correlator in incoherent light, the holographic correlator, hybrid opto-electronic correlator, the electro-optic correlator for microwave interferometry and a window-based optical correlator. I represented all these types in Figure 1.

### 2.1. JTC Optical Correlator: Theory, Types and Main Application (Cryptography/Security)

The most extensively researched type of correlator today seems to be the joint transform correlator (JTC). This section details the characteristics of JTC correlators, of the cryptosystem based on JTC and various types proposed in recent papers.

The importance of information security nowadays cannot be overestimated. Data need to be protected in every institution (industry, research, healthcare, and governments), in personal identification documents (identity card, passport, credit card) and in information transfer and processing. To achieve data protection against intrusion, counterfeit or forgery, various methods have been proposed to encrypt and decrypt the data [23]. Such methods could be classified into three major categories: optical, digital, and digital methods inspired by the optical ones. Five of the most used techniques for increasing the security of information are: cryptography, steganography, watermarking, methods based on optical signal processing and, more recently, quantum cryptography techniques [24].

To encrypt information and for other applications in watermarking, secure identification, recognition or verification, optical techniques have been used as an efficient solution, due to advantages such as the inherent parallelism of optics, the large capacity of information transmission, robustness, flexibility, and the high speed of the information processing [25,26,27]. Specifically, optical encryption uses light propagation through an optical setup, to encode the information. The process can be reversed by using an encryption key [28].

Among the optical encryption solutions, the most common is the DRPE (double random phase encoding), developed in 1995 by Réfrégier and Javidi. The setup involves a 4-f classical processor or a JTC, that performs both the encryption and decryption [29].

As advantages of the DRPE method, one can mention the possibility to process the information in parallel, at a high speed, together with the various freedom degrees of the encryption (due to properties of the optical fields such as coherence, wavelength, polarization, modulation etc.) that enable different setups of optical encryption with increased security [28,30].

DRPE presents, however, several drawbacks: a complex ciphertext is difficult to record; the precision of the alignment must be very high; the fabrication of the phase conjugation mask is challenging; in the decryption process, one needs the precise complex conjugate of one of the RPMs employed as a key [29,31]. Moreover, DRPE cryptosystems are prone to attacks such as KPA (known-plaintext attack), CPA (chosen-plaintext attack) and COA (ciphertext-only attack) [32].

To overcome the drawbacks of the classical encryption techniques mentioned in the previous subsection, the joint transform correlator (JTC) was proposed as an alternative solution. Thus, the architecture of a cryptosystem based on JTC has become the preferred choice in most applications [29], due to advantages such as the possibility of using without an extremely precise alignment of the optical components (because the object and the key are both in the same plane); the elimination of the requirement of a complex-conjugate of the encryption key for decryption; the identity between the JPS (joint power spectrum) and the ciphertext [25,28,33].

**Figure 2:**
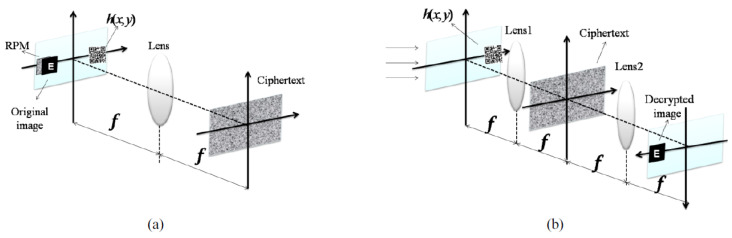
The scheme of a cryptosystem based on JTC. Figures (**a**,**b**) present the encryption and decryption processes, respectively [33].

The scheme of a JTC cryptosystem, including the processes of encryption and decryption, is presented in Figure 2.

The encryption part begins with the bonding between the initial image, f(x,y), and the RPM, denoted with ψ(x,y). Secondly, a spatial light modulator (SLM) displays, side by side, the product between f(x,y) and ψ(x,y), at coordinates (−a, 0), and the key mask, β(x,y), at coordinates (a, 0). The placement of the SLM is in the input plane of a FTL (Fourier transform lens). In the example in Figure 2, the input plane also includes a phase distribution h(x,y), when a QR code is used for encrypting multiple images. In the focal plane of the same FTL, the joint power spectrum (JPS) is recorded. This will be the ciphertext, J(ξ,η). In the decryption part, the first FTL has, in its input plane, at coordinates (a, 0), the key mask. Then, in the focal plane of the same FTL, the multiplication between the key mask and ciphertext is performed. Afterwards, a second FTL is placed, and in its focal plane the recovery of the plaintext is finally obtained [29]. Thus, the encrypted image consists in a distribution of the intensity in the Fourier plane, whereas the decrypted image results after using the same key as for the encrypted part [31]. However, there are still some downsides of using cryptosystems based on JTC. The main two are the noise, created in the JTC setup, that significantly reduces the decrypted image quality, and the problems regarding the security, that make these cryptosystems prone to several attacks (the same as for DRPE). The latter drawback is due to the linearity of the encryption setup in the conventional scheme (the initial image and the ciphertext are linearly related). Although various proposals decreased their vulnerability to attacks, by eliminating the linearity, most of these solutions also increase both the complexity and the price of the system, which is a disadvantage in practical applications [29]. Other proposals lead to a simplification of the encryption part, mainly the optical setup, and respectively to an improvement of the decrypted image quality [31]. An alternative to the JTC cryptosystem is the JFTC (joint Fresnel transform correlator) one, that retains the properties of the JTC cryptosystem with additional benefits, such as the possibility of using more encryption keys, based on the position of the key mask in the input plane of the FTL and the diffraction distance [30]. Another extension of the JTC cryptosystem is in the Fresnel domain, by using phase-shifting interferometry, enabling the processes of binary data encryption and decryption to take place at a high speed and the operation in a simple and robust setup [34]. The JTC cryptosystem was also extended to the fractional Fourier and Gyrator domains, respectively, having as a result an improved quality of the images and an increased security against attacks [35].

**Figure 3:**
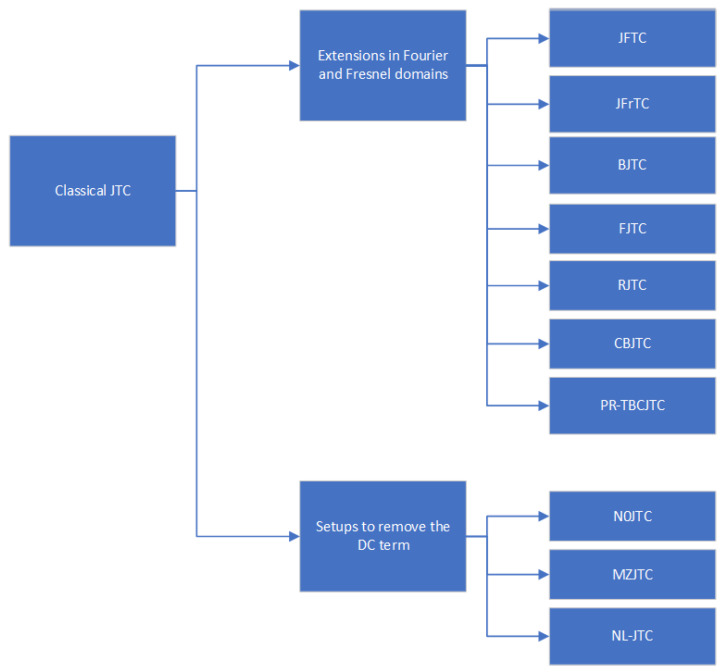
Overview of various alternatives to the classical JTC, where: JFTC—joint Fresnel transform correlator, JFrTC—joint fractional Fourier transform correlator, BJTC—binary JTC, FJTC— fringe-adjusted JTC, RJTC—reference phase-encoded JTC, CBJTC—compression-based JTC, PR-TBCJTC—photorefractive two-beam coupling JTC, N0JTC—nonzero-order JTC, MZJTC—Mach–Zehnder JTC, NL-JTC—non-linear JTC.

Various architectures have been proposed in the last decades to improve the characteristics of the classical JTC, as shown in Figure 3.

The JFTC is a version of the JTC that is based on the Fresnel transform, with the information stored as a distribution of the intensity, named JFPD (joint Fresnel power distribution). It has the advantages of including an additional security parameter, namely the propagation distance, in free space, between the two end planes (input and output) and the simplification of the setup, that does not require a lens as the classical JTC [36].

The JFrTC is another JTC alternative, also used mainly in cryptography applications. It benefits by the addition of several new security parameters as compared to JTC and JFTC: polarization, wavelength, in-plane shifting, key rotation and, in more recent developments, the fractional order of the transform. However, as compared to the JFTC, it includes a lens, as in the classical JTC setup [36].

The BJTC is based on the binarization of the JPS (joint power spectrum) by using the Fourier plane nonlinearity. As advantages of the BJTC one can mention an increased ability of discrimination of the output correlation peak, with a high intensity and a narrow width of the correlation. The main disadvantage of the BJTC is constituted by the false alarms generated by the harmonic correlation peaks of higher orders, the problem being more severe when the input plane includes more than one target [37].

The FJTC is based on the multiplication, in the Fourier plane, of the JPS by a filter with real values. It reduces the problem of the false alarms of BJTC, because it does not create, in the output plane, harmonic correlation peaks of higher orders. On the other hand, false alarms may still appear, when the input scene includes several identical targets and other objects [37]. For face recognition applications, the FJTC (fringe-adjusted JTC) has been used, in various configurations and subtypes, with a more recent proposed version in the last years: the 4-PSK WSJTC (four-phase shift-keying wavelet-filtered-based shifted-phase encoded JTC). The 4-PSK WSJTC successfully eliminates the DC term and the problem of autocorrelation when the input scene consists in multiple objects, identical or similar. The wavelet transform is used for obtaining a more detailed information of the analyzed objects [38,39].

The RJTC improves significantly the detection of multiple identical targets in the input plane, by avoiding the problem of false alarms. Moreover, as compared to other variations of the JTC, it creates for each target only one correlation peak and thus uses more efficiently the space-bandwidth product [37].

A multi-step JTC was also proposed, proving successful in eliminating the DC term and the false alarms due to the presence, at the input, of identical objects. It is a concept used with efficiency for DNA sequencing [37].

Developed mainly for applications in tele-ophthalmology, the CBJTC is an extension of the JTC architecture characterized by the compression of both images (target and retinal reference) in a JPEG (Joint Photographic Experts Group) format [40].

To improve the characteristics of the conventional JTC architecture, the PR-TBCJTC was also introduced. It presents five main advantages as compared to the classical JTC: a reduced number of false alarms, leading to a more efficient detection; an operation in real-time, adaptable; an improvement of the SNR (signal-to-noise ratio) by means of dynamic range compression techniques; an enhancement of the correlation peaks intensity at the output; a simultaneous improvement of all the metrics involved [41].

**Figure 4:**
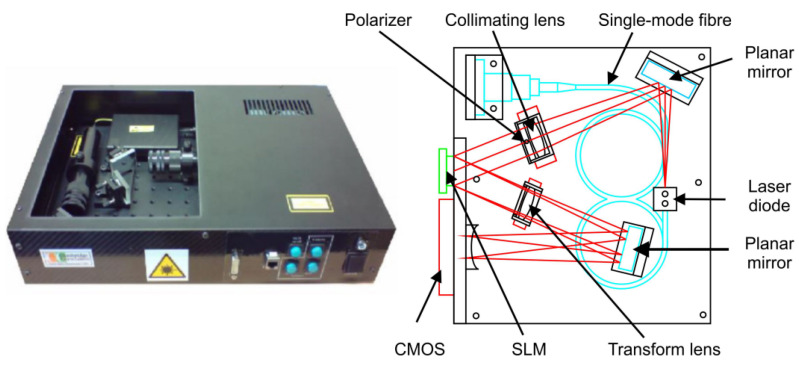
The Cambridge optical correlator [44].

Practical versions of the JTC correlator have also been developed, such as the Cambridge correlator, associated with the FOE (Fourier Optics Experimenter) software, used for pre-processing the images captured by a video camera, that are afterwards compared by using the correlator. This type of correlator, developed at Cambridge University (thus its name), is presented in Figure 4. It includes a laser diode, SMF (single-mode fiber), collimating lenses, planar mirrors, a polarizer, a spatial light modulator (SLM), an analyzer and a CMOS sensor. The arrangement of the components in a shape like the letter W reduces the distance of electric signal transmission and the processing time. The main disadvantage of this correlator is its size, although in current applications its size is reduced at approximately half of its initial size [42,43,44].

Several other types of JTC were proposed, to overcome a significant disadvantage of the classical JTC setup: the presence of the DC term (created in the correlation plane by the target and reference images auto-correlation sum), that reduces the performance of the device and must be therefore minimized or eliminated. To do that, the N0JTC was proposed, using the technique of phase-shifting (the 1997 version) or the strategy of JTPS (joint transform power spectrum) subtraction (the 1998 version) [45]. In 2002, an improved JTC version was developed: MZJTC. As compared to the N0JTC, the MZJTC correlator can remove directly, in a single step, the DC term. Thus, it avoids the need to store, before this step, the Fourier spectra for the target and reference images.

The JTC family also includes the NL-JTC, based on nonlinear materials, having the advantages of a reduced latency and better performance as compared to the classical JTC and other JTC architectures, due to the elimination of the need to interface a CCD camera with a SLM (that involve a multiple-step process which creates noise and increases the time and cost of the system). In an NL-JTC, for controlling the contrast between the correlation peaks at the output, the Fourier plane is provided with a nonlinear intensity threshold. One of the possibilities for obtaining a NL-JTC is to benefit from the nonlinear process of four-wave mixing, to create a JTC based on this process [46].

To conclude, there is a wide palette of JTC subtypes proposed in the last decades, in a race towards achieving the best properties in various applications. The JTC remains probably the most researched optical correlator today. However, other researchers concentrated, instead, on the use of the other important correlator, the VLC, also with significant results, as depicted in the next sub-section of our paper.

### 2.2. VLC Optical Correlator: Theory and Main Application (Face Recognition)

The VanderLugt correlator (VLC) has proved to be successful mainly in face recognition applications, where two images (a target and a reference) can be compared, by using a lens able to perform the Fourier transform (FT). Other advantages of the VLC are the exceptionally fast time required by the entire process, the high SNR (signal-to-noise ratio) and the large values of the space-bandwidth product, together with characteristics of the matched filter such as robustness, efficiency, location accuracy and high capability of discrimination. On the other hand, this type of correlator also presents some drawbacks: the alignment of the components in the setup must be extremely precise; it is more complex than the JTC; the matched correlation filter is very sensitive both to rotations of the input image and to changes of the scale size [20,22,47,48].

**Figure 5:**
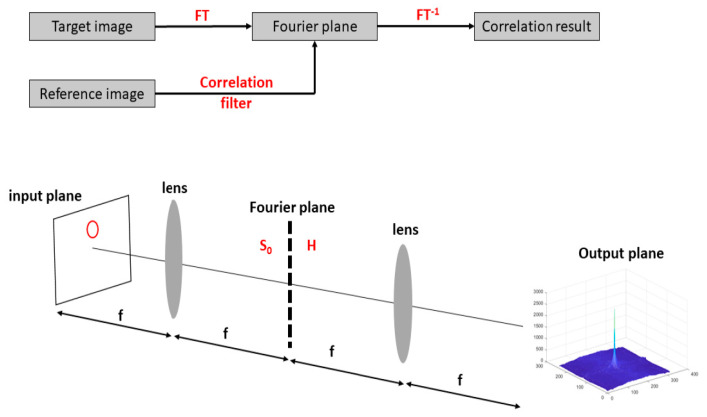
The structure diagram of the VLC optical correlator [6]. The circle in the input plane represents the face image. $S_{0}$ is a lens in the Fourier plane and *H* is a filter in the same plane.

The setup of a VLC correlator is shown in Figure 5. It includes three planes (input, Fourier, and output) and two convergent lenses that separate each two planes. As stated above, two images are compared. One is the target, placed in the input plane, whereas the other one is a reference. A lens performs the FT of target image spectrum, that is multiplied, in the Fourier plane, by a matched correlation filter, obtained from the reference image. A second lens is then used, to apply an inverse FT to the multiplication result. Finally, in the output plane a correlation peak is obtained, together with other smaller peaks. The degree of similitude between the target image and the reference one is given by measuring the central correlation peak, having the highest value of the PCE (peak-to-correlation energy), and comparing this value with a fixed threshold [1,6,21,49].

To evaluate the correlation between the target and reference images, the PCE parameter is used. It can be determined as the energy of the central correlation peak (the highest in the output) divided by the total energy in the output plane (Equation (1)):

(1) $$\mathrm{PCE}=\frac{\sum_{i,j}^{N}E_{highestpeak}\left(i,j\right)}{\sum_{i,j}^{M}E_{total}\left(i,j\right)}$$

where *N* denotes the size of the spot obtained at the output, corresponding to the highest correlation peak, and *M* refers to the size of the output correlation plane [49].

Whereas the JTC progressed through the proposal of various new architectures with better performance than the original one, the research on the VLC concentrated more on various novel proposals of a better matched filter, while keeping the architecture unmodified. Such alternatives to the classical matched filter are: POF (phase-only filter); correlation filters with encoding of both amplitude and phase; trade-off correlation filters; ternary encoded filters; correlation filters with syntactic discrimination; optimal filters etc. [41]. The names of seven of the most used filters in VLC correlators are: CMF (classical matched filter), CTMF (complex ternary matched filter), POF, BPOF (binary POF), QPF (quad phase filter), MACE (minimum average correlation energy filter) and the composite filter [1]. Among these filters, the POF has been used frequently, for its high robustness and discrimination ability, due to the better quality of the image obtained by using the phase information instead of the one given only by the amplitude [50]. However, regardless of type, most of the matched filters have the advantage of being relatively easy to implement. However, they have the drawback of introducing, as compared to the JTC, an additional processing step, required for phase information extraction [41]. The quest for finding the best matched filter has been directed towards solving some problems or improving some characteristics of the VLC correlator, such as: better discrimination; lower rate of false alarms; improved SNR; obtaining the invariance of the correlator to distortions caused by scale, rotation, or other changes [41].

The VLC correlator has found a wide range of applications, mainly for pattern recognition (most often, face recognition), from industry, sports, medicine, and the military domain to various applications of person recognition for achieving an improved security in buildings and other locations and to manage places that easily become crowded or are sensitive, such as official buildings, stations, or airports [10,49]. In face recognition systems, the VLC correlator generally performs the third out of the four steps of the process, that includes: feature extraction, preprocessing, feature extraction (that can be done with the VLC) and classification [51]. The VLC correlator can also present better results as compared to other methods when face recognition is more difficult due to head rotations [52]. Other challenges that increase the difficulty of recognizing the faces of people are variations of pose, facial expression, light, changes due to aging and others [51]. With carefully chosen characteristics, a VLC correlator can alleviate or even eliminate some of these difficulties. Details on these techniques and applications of the VLC proposed in the recent years will be given in a dedicated section.

### 2.3. Other Types of Optical Correlators

The JTC and VLC correlators are the most frequently used types of optical correlators both in research and applications. However, there are also other versions of the device that have been successfully used in several domains: the optical correlator in incoherent light, the holographic correlator, the hybrid opto-electronic correlator, the electro-optic correlator for microwave interferometry, the window-based optical correlator and the single micro- or nanowire correlator. This section briefly presents their main characteristics and functioning principle.

#### 2.3.1. Optical Correlator in Incoherent Light

The first implementation of a correlator working in incoherent light was proposed by Lohmann in 1968. As opposed to the VLC correlator, where the correlation signal is formed by the amplitude, in the incoherent correlator it is formed by the intensity [53].

The main advantages of this type of correlator are its invariance to translations of the analyzed image and its robustness: it is relatively unaffected by defects, noise, or errors, such as the presence of scratches or dust on the optical surfaces, or errors due to imperfect alignment between the components of the setup, as opposed to the VLC correlator, where the alignment is especially critical. On the other hand, the main drawback is that is relatively unable to discriminate between slightly different images [10].

A variation of the classical correlator in incoherent light is based on the DMD (digital micromirror device) modulator. It is a holographic correlator (as in Section 2.3.2) but using incoherent light. It benefits from the advantages of DMD modulators, such as the possibility to obtain images with a very good quality, with a resolution up to 1920 × 1080, with a pixel matrix of a small size (approximately 13 μm) and a binary image change rate at a 32 kHz frequency. There is no need to perform phase modulation as a function of the wavelength, because DMD modulators perform amplitude modulation by using the light reflected from the micromirrors of the matrix. In the setup, incoherent light from the source is directed, by means of a single lens, on the DMD modulator, where the Fourier hologram is displayed. In the output plane, the correlation result is obtained [53]. Another alternative is to use a diffractive correlator, that also includes, additional to the elements in the classical incoherent light correlator, some periodic diffractive elements, such as diffractive gratings, capable of splitting the incident light into several secondary beams. This method can find applications in several correlation schemes, with good results in determining the correlation product of the intensity spectra [54,55].

Another possibility of using incoherent light in an optical correlator is to use an architecture of a dispersive correlator. Because incoherent light is used instead of the coherent one, chromatic effects may arise, that need to be compensated by using additional elements in the setup. Thus, objects can be recognized by using either generated or scattered direct radiation, that enables a high processing speed and a reduction of the cost, weight, and size of the system, but on the other hand only the spatial characteristics of objects can be recognized, which is impractical, because in many cases the shape of an object is not enough for its identification. Therefore, one needs also to know the radiation spectrum corresponding to the object, i.e., both the spatial and spectral characteristics. The correlation signals are created by the interaction between the radiation corresponding to the target object and Fourier holograms, used as spatial filter memories. The hologram includes a spatial pattern of the reference object, with copies of different sizes of the reference. Each component of the input radiation gives a correlation signal and the total distribution of these signals, at the output, offers the final correlation result [56]. Holograms are also used in the holographic correlator, that I analyze in the next sub-section.

#### 2.3.2. Holographic Correlator

**Figure 6:**
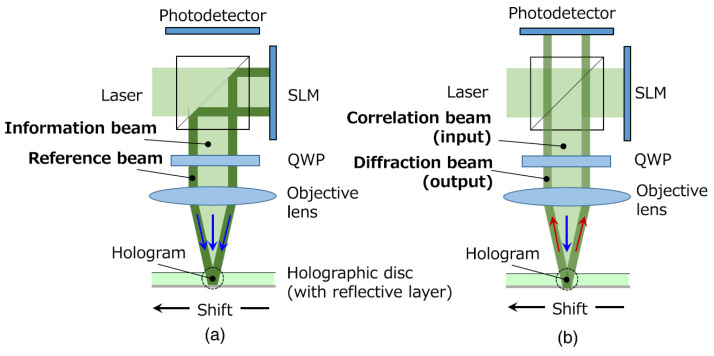
The holographic optical correlator: (**a**) the recording part; (**b**) the correlation part. QWP: quarter wave plate; SLM: spatial light modulator [57].

The technique of holographic optical correlation involves a holographic disc, where information about the reference image is recorded as a hologram, and the irradiation on it of the target image data. In the holographic correlator, two beams of light (information and reference) are combined in one, on the same axis. The property of the hologram of shift selectivity is used for performing a multiplexed recording of the combined beam. Figure 6 presents both the recording method (using the information and reference beams) and the correlation method (using the correlation and diffraction beams).

If the holographic disc rotates with a speed R (expressed in rotations per minute, rpm) and one denotes with r the radial position of the disc to be correlated (expressed in mm) and with d the shifting distance when multiplexed recording is performed (expressed in μm), one can express the correlation speed (expressed in frames per second) of the holographic correlator using the expression in Equation (2):

(2) $$V=\frac{R}{60}\cdot \frac{2\cdot \pi \cdot r}{d}$$

From Equation (2) it can be remarked that the correlation speed can be increased if the rotation speed R of the holographic disc is increased (by means of an improved hardware design), and the shifting distance d is decreased [57]. The increasing of this speed is the goal of some more recent research efforts. In such attempts, a variation of this type of correlator was proposed: the coaxial holographic optical correlator, that achieved a speed of 143 Gbps [4]. The optical setup in this case has similitudes with the technique of disk control, employed in traditional optical disks. The technique uses an HVD (holographic versatile disk), developed in 2005 as a holographic memory that uses a coaxial holographic optical setup, including two lasers with different values of the wavelength (e.g., 532 nm and 650 nm, respectively). The first laser is used for recording the hologram and for obtaining the optical correlation, whereas the second has a role in the servo control method. Such a system has proved good results in terms of stability and correlation efficiency [4]. Other versions of the optical correlator also include an electrical part, thus resulting in hybrid opto-electronic correlators.

#### 2.3.3. Hybrid Opto-Electronic Correlator

As opposed to holographic correlators and to the classical JTC and VLC setups, the hybrid opto-electronic correlator (HOC) benefits from better materials employed for recording: films of photorefractive polymers are replaced by materials with photodetection properties [8]. Moreover, as compared to the VLC correlator where the comparison of two images can be achieved by employing a holographic filter, the HOC correlator increases the speed by eliminating the slow process of recording required by the filters. As compared to the JTC correlator, that also eliminates the recording process of filters, the HOC correlator uses improved materials, that do not require exposure to high voltages that might damage them beyond repair. The HOC correlator also possesses the advantage of being invariant to shift changes and, in more recent developments, architectures of the HOC correlator were developed that are also invariant to scale and rotation changes [58].

A proposed HOC includes SLMs, photodetectors, circuits for phase stabilization and VLSI chips. The photodetectors are used for recording the data about the amplitude and phase of both target and reference images, by means of the interference with planar waves. The reliability of this type of correlator was recently demonstrated experimentally [8]. Another type of correlator that is not all-optical is the electro-optic correlator used for microwave interferometry. It can correlate a high quantity of signals in the microwave spectrum, in a large range of frequencies and achieves a significant complexity reduction. It was proposed as a part of an interferometer prototype, in CMB (cosmic microwave background) applications, for the characterization of the lower frequency bands (approximately between 10 and 12 GHz) of the CMB [59]. The hybrid opto-electronic correlator was shown to achieve comparable results as the holographic correlator, without the need of using holograms [58].

#### 2.3.4. Window-Based Optical Correlator

An alternative to the classical types of optical correlators is also represented by the window-based optical correlator (WOC), with an interesting application in the alignment of DNA sequences, that consists in the comparison of DNA sequences characters with a reference database. The WOC correlator can perform this comparison, by selecting windows (several intervals of the DNA sequence partitions), extracting the correlation peaks between the windows and the sequence of reference, and concluding which are the regions having a similarity score that exceeds an imposed threshold [16,17].

The recent applications of all types of optical correlators described above will be detailed in the following section.

## 3. Recent Applications and Implementations of Optical Correlators

Most research papers in latest years in the field of optical correlators involve the use and application of JTC correlators, mainly for security purposes, but also for image recognition and some medical and spatial applications.

### 3.1. JTC Optical Correlator Applications

Cryptosystems based on the JTC correlator have been extensively used recently, with various proposals of systems for encryption and authentication that are resistant to attacks and eliminate noise problems. Below, I detail such JTC cryptosystems that proved successful in such applications.

#### 3.1.1. JTC Cryptosystems for Security Applications

Among the earlier contributions to JTC cryptosystems, one can mention the first successful color encryption, demonstrated experimentally in a JTC in [60]. It was achieved in a setup where three images were used, with different wavelengths (in the cyan, green, and red spectra, on 520, 556 and 647 nm, respectively), each wavelength being used as key, together with the RPMs, for both encryption and decryption. The contribution is a step forward as compared to previous monochromatic illumination systems, where the input image data were lost [60]. JTC architectures can also be used for encryption of movies, the first successful demonstration being reported in [61]. The proposed technique was also extended for multiplexing in one single package three different movies. For encryption, a Mach–Zehnder interferometer was used, with one arm represented by the JTC, and the other arm with the wave employed as reference [61].

The importance of security in optical cryptosystems is essential. To enhance the security of a JTC-based setup of optical encryption, a scheme with confused ciphertext was proposed [29]. As compared to the traditional JTC cryptosystem, it avoided its linear character, by a process in three steps: the decomposition of the original image in two phase-only masks; the transformation of one mask into JPS (joint power spectrum); the use, as final ciphertext, of the JPS confused with superposition and rotation operations. The setup demonstrated an enhanced robustness against several attacks, mainly the one based on the IFT (iterative Fourier transform), a low cost, an increased simplicity and was validated by numerical simulations [29].

Concerning the same concerns about security, a linear cryptosystem was also developed, based on JTC, to analyze the effects of KPA (known-plaintext attacks) [62]. Operations to eliminate noise were applied to recover the input image of the JTC cryptosystem with a very good quality. This process, however, makes the ciphertext linear with respect to the original image, thus lowering the security of the setup. By performing a KPA, two pairs of known plaintext—ciphertext were randomly selected to calculate the encryption key. The ciphertext was sampled, by using the Shannon–Nyquist sampling theorem, to reduce the cost of computation for the procedure of attack. The validity of the KPA was demonstrated by means of numerical simulations [62]. The ability to resist several attacks was recently enhanced in another JTC system proposal [63], using the cropping operation for resisting attacks instead of a method of attack as in other papers. Simulations confirmed that the system can resist different attacks (KPA, COA, CPA), without losing the image quality at the output or increasing the complexity of the system, if 90% of the ciphertext is transmitted and the rest is cropped. The increase in the security of the system was confirmed by both simulations and experiments [63]. The reduction of noise, specifically speckle noise, was also achieved in another paper based on the JTC setup, where the input was arranged in an innovative way, benefitting fully from the available space on the SLM and a rearrangement of the input objects, without increasing the complexity or the hardware resources of the system. Thus, an improved quality of the object obtained at the output, after the reconstruction, was demonstrated [25].

More recently, another method was proposed to reduce noise in a JTC system, by using a deep learning technique that consists in adding dense modules into the generated network, thus improving its performance and the possibility to reuse feature information. The proposed system proved a better results for the mean-square error (MSE), peak signal-to-noise ratio (PSNR) and structural similarity (SSIM) as compared to previous algorithms of noise elimination [64].

Another recent implementation of the JTC cryptosystem was based on the PST (phase-shifting technique) [65]. It avoided two significant issues of usual JTC cryptosystems that involve PST: the requirement for the user to know information about the plaintext before the process of decryption and the possibility to encrypt only binary plaintexts, not also colored or gray-scale images. The proposed algorithm includes an operation of pre-processing, based on an intensity modulator, with a PC (photon-counting) operator and employing the method of histogram equalization, that achieves, as input for the cryptosystem, a binary distribution of the plaintexts that is limited by the photons, thus increasing the security of the system (as the input information is not visible in the decoded images) and enabling the use of the unchanged optical setup for encrypting color and gray-scale images additional to binary ones. Moreover, an operation of post-processing was also included, to extract from the encoded images information about phase and to obtain the authentication of the decoded images. The algorithm was validated by numerical simulations [65].

**Figure 7:**
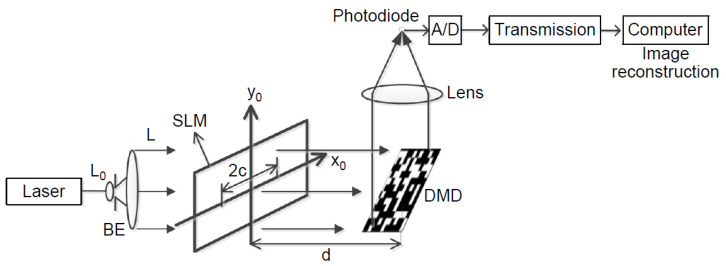
Proposed setup of a system of encryption of compressed images using PST and a JTC. Here, L: light; BE: beam expander; SLM: spatial light modulator; DMD: digital micro-mirror device [66].

The PST interferometric method was also used in another proposal of encryption of optical images, that employs the JTC and, additionally, compressive sensing techniques [66]. First, a method of binary scrambling is used for a permutation of the object image. Secondly, PST is employed to encrypt and register, as holograms, the field of the permutated object on the JTC. Thirdly, a compression step takes place for both the key and the encrypted images. Finally, the proposed algorithm is used to reconstruct and decrypt the original image. The setup is presented in Figure 7. The efficacy and suitability of the method for secure image transmission were demonstrated by means of numerical simulations [66].

To improve the quality of decrypted images, a JTC was proposed, where gray-scale images are binary encoded [67]. The system uses in the process binary encoded images instead of the gray-scale unencoded ones, since they are not sensitive to non-saturation noise, and a median filter to also eliminate the saturation noise. As compared to the classical JTC setup, this method found an improvement of the coefficient of correlation between the initial image and the decrypted one, from 0.8237 to 0.9937. Moreover, the speed of the encoding process proved much higher as compared to QR encoding. The approach was confirmed by experimental results [67]. The same group also simulated the processes of encryption and decryption in a JTC cryptosystem with gray-scale images, where complex amplitude modulation was performed by single-pixel hologram encoding using a phase-only SLM. The proposal demonstrated an improved efficiency over other binary methods for encoding gray-scale images [68].

To increase the accuracy of sample analysis, both in materials science and medicine, as compared to traditional optical detectors, able to receive information only about the intensity and not also of the phase of the light beam, a system for encrypting images was recently proposed, based on both the JTC and the method of ptychography [69]. At the encryption stage, the scanning movement of the probe (from the ptychography iterative engine) is used to divide the initial image into different parts. Thus, several encrypted images are obtained that are decrypted and reconstructed at the output, using both the JTC and ptychography techniques. As advantages of the method, one can mention a simplified setup and functioning of the system, due to less strict constraints concerning the alignment and arrangement of components; a better security, due to the presence of numerous keys, that must be all correct for the restoration of the original image; the elimination of optical interference in the system. The feasibility of the method was proven by numerical simulations [69].

JTC correlators have been also used for encryption and authentication systems with multiple users. In a recent implementation [34], such a system was proposed, where during the process of encryption the plain-text is encrypted by means of the fingerprints of several users, whereas at the onset of the process of decryption the authentication of each user is performed, thus eliminating the access of unauthorized ones and increasing the system security. The paper also demonstrates that the JTC is successful in avoiding the overlap between images at the output of a system with two encrypted images and multiple users, if the input images are separated by three times the side length of a square image. The feasibility and efficiency of the system were demonstrated by simulation results [34]. Another setup that enables the authentication of two users was proposed by another group of researchers using a nonlinear JTC [35]. The system needs from each user one digital fingerprint and their authentication is performed simultaneously. In addition to the two fingerprints, three RPMs are used, and these five security keys permit a better resistance of the system as compared to previous proposals, even when attacks (such as plain-text or brute force) are performed on it. Numerical experiments with digital fingerprints validated the approach [35]. Two users can also use a proposed secret sharing scheme, based on both compressive sensing techniques and the JTC setup [70]. Plaintext is encrypted by using as keys the fingerprints of the two users, and the decryption is possible only if the authentication of both fingerprints is successful. To avoid the linearity between ciphertext and plaintext, compressing sensing is used, that can resist attacks performed on the JTC. The system was validated by numerical simulations [70]. Encryption of multiple images into a single ciphertext was also obtained with a JTC by rotating the spiral phase mask (SPM), more resistant to attacks as compared to the traditionally used random phase masks (RPMs) [26]. Thus, when the ciphertext is decrypted, instead of using several key masks, it is necessary only to rotate the SPM to the specific angle that corresponds to the plaintext. Both numerical simulations and experiments demonstrated the validity of the method and the resistance of the system to two types of attacks (differential and occlusion) [26]. Multi-channel images were also encrypted simultaneously into a single ciphertext, without additional hardware or any increased complexity of the system, using a JTC setup [71]. The ciphertext can also be used for recovering the initial images if the correct keys are employed. The problem of cross-talk between images was solved by using optimized phase masks reconfigured on a single SLM, to restrict the JPS to a certain area, and linear phase shifts, to control the single JPS position and split the multiple JPS. The device can transmit a large quantity of encrypted data. The proposed system was validated by both simulations and experiments [71].

JTC cryptosystems have also been proposed based on QR (quick-response) codes and an information container, for security applications, since the first successful implementation of a QR code as container in a JTC system, in 2014 [27]. In one such recent application [33], a JTC system able to encrypt multiple images was introduced, using as key masks QR codes, obtained from conversion of various texts, in English, Chinese and even symbols. Thus, instead of transmitting the entire key mask, only the text necessary for the key generation is transmitted. The method was validated both by simulations and experiments [33]. Another application achieved the encryption of data based on ghost imaging, using a customized information container and the XOR (exclusive OR) operation, in two steps, with an increased security and recovery of the initial data without losses, validated by simulations [72]. The same research group also proposed a method of encryption for binary images in a JTC setup, using QR codes and the RLE (run-length encoding) algorithm [73]. The initial binary image is compressed using RLE, scrambled with a chaos-based method, transformed into a QR code (with the same size as the original image, for the first time) and, finally, encrypted in the JTC. The improved security (due to scrambling), noise tolerance and feasibility of the method were demonstrated by means of simulations [73]. To avoid speckle noise in the output of JTC cryptosystems, a customized information container was also proposed by another group [18], having a low spectral bandwidth, a noise tolerance level easily controlled, and a quick and easy readability, enabling an increase of the quantity of degradation-free recovered data, a significant reduction of system complexity and an increased tolerance to information losses. The method was validated by both experiments and simulations [18].

The JTC cryptosystem was also extended in three dimensions [74]. Data on any 3D object can be thus encrypted, where the encryption key is represented by a second 3D object. Thus, this setup can protect 3D data. The method was validated by experiments, with a setup using a DPSS (diode pumped solid state) laser, a CMOS camera and, as opposed to traditional 2D JTC setups, no SLM [74].

Various other architectures of JTC cryptosystems have been proposed, with different modifications to improve its performance. Recently, a novel scheme of optical encryption was introduced, based on a nonlinear JTC, to implement the DRPE (double random phase encoding) technique [19]. The input plane contained two random phase masks (RPMs), as in the usual DRPE setup, used as security keys, and the image, fully phase-encoded (with a phase-only SLM), that is encrypted. For the JTC to give identical results as a DRPE setup with a classical 4-f processor, the JPS (joint power spectrum) of the JTC was modified by the addition of nonlinear operations, to obtain images with better quality. The feasibility of the system was confirmed by both simulations and experiments [19].

Recently, novel implementations of JTC asymmetrical optical correlators have also been proposed, using the SSPM (structured spiral phase mask) instead of RPM, and the RSA (Rivest–Shamir–Adleman) cryptographic algorithm. Such proposals are efficient in overcoming the vulnerability to attacks of the JTC cryptosystems due to its inherent linearity, with the same key used both in encryption and decryption stages. In an asymmetric cryptosystem, the keys for encryption and decryption are different and cannot be deduced one from the other. Two recent implementations used SSPM masks, the RSA algorithm, and an operation (either image multiplication and division, or image superposition-subtraction) to obtain novel asymmetric cryptosystems based on the JTC. The image multiplication and division operation was created, for the first time, by the use of a trapdoor function. The SSPM mask was preferred instead of the more frequently used RPM mask, because the latter is more difficult to replicate with high accuracy (mainly its complex conjugate) and requires for its generation a large amount of data, increasing the risks of being intercepted by an attacker or of being lost. The proposed methods were validated both by simulations (in MATLAB) and by preliminary experiments, with a setup including a He–Ne laser and a phase-only SLM [75,76].

Table 1 presents a comparison of the parameters chosen for the components of the optical setup in various proposals of JTC cryptosystem architectures.

Whereas the above implementations refer to the classical JTC cryptosystem, based on the Fourier transform, with or without additional modifications, the next section presents recent variations of this architecture where the Fourier domain is replaced by Fresnel, Gyrator, Fractional Fourier, or Collins domains, respectively.

#### 3.1.2. Variations of JTC Cryptosystems (Fresnel, Gyrator, Fractional Fourier, and Collins) for Security Applications

**Figure 8:**
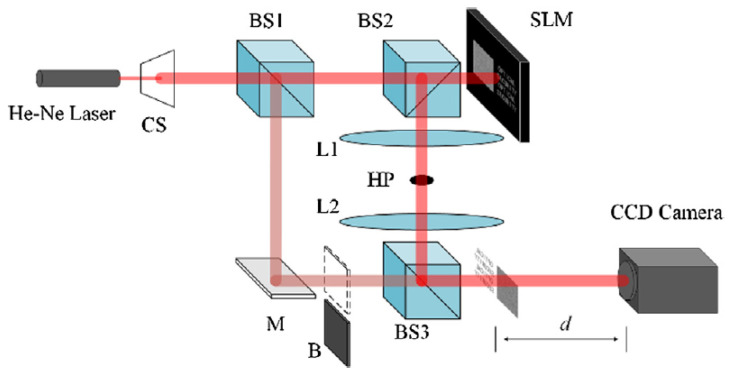
Encryption system based on a joint Fourier transform correlator (JFTC). Here, CS: collimation system; BS1, BS2, BS3: beam splitters; SLM: spatial light modulator; L1, L2: lenses; HP: high-pass filter; M: mirror; B: baffle; CCD: charged-coupled device [30].

Vilardy et al. [77] proposed the extension of the nonlinear optical JTC cryptosystem to the Fresnel domain, in a simplified optical setup, with a random phase mask as key, no lens and beam-splitting, and a nonlinear operation introduced in the encrypted function. The property of shift-invariance of the mask to lateral displacements during the process of decryption was preserved, and the robustness against CPA and KPA attacks was shown to increase [77]. More recently, another system for optical image encryption, based on a JTC using the Fresnel transform, was experimentally studied [30]. The proposed system achieved an improved reduction of noise in the decrypted image and a significantly better quality of this image, by means of an optimization of both the random phase and key masks. The experimental setup is presented in Figure 8.

The JTC based on the Fresnel transform was also researched in terms of computing occlusion (partial) and noise (additive and multiplicative), to evaluate their influence on the decrypted image quality [78]. As compared to the setup in [30], there is no lens present. The robustness of the system was determined by determining the RMSE (root mean square error) between the original and decrypted images, in conditions of noise or occlusion. The system still presents acceptable performance when up to a quarter of the encrypted image is occluded [78].

Another JFTC architecture was proposed for performing optical image watermarking [66]. Watermarking techniques have been introduced for images and, in other applications, for audio recordings [79]. In [66], a novel technique of optical image watermarking was performed, by embedding a secret image into a host one, by using the JFTC, followed by a compression of the watermarked image. The feasibility of the system was confirmed by numerical simulations [66].

For optical image encryption, a JFTC with double optical wedges was also introduced [80]. The setup achieved an improved quality of the decrypted image as compared to JTC cryptosystems, due to the significant reduction of noise during decryption. The system also proved an improved resistance to attacks and its reliability was confirmed by simulations [80]. JFTCs have also been used with multi-channel images. A method was proposed to obtain a parallel encryption and hierarchical retrieval of such images with a JFTC [81]. It used the operation of linear phase shift and the algorithm of phase retrieval, obtaining improved phase masks and encryption of multi-channel images in a single ciphertext, that is also used for the retrieval of the original images. The security was improved as compared to JTC cryptosystems by using two additional keys: the key masks position and the Fresnel diffraction distance. The feasibility of the system was confirmed by simulations [81].

Another implementation of the JFTC was performed with a Billet split lens (BSL) [82]. The split width of the BSL can be adjusted, thus offering the possibility to control the joint power distribution of the Fresnel transform in terms of size and recording position. To eliminate noise, the text was converted to a QR code and used as a plaintext. A spiral phase mask (SPM) was used instead of a RPM, for an enhanced concentration of the ciphertext distribution. A complex secret key (CSK) was designed by using the SPM and a QR code filter. Both simulations and an experimental setup validated the cryptosystem [82]. A JFTC architecture was also proposed and analyzed in an experimental setup by [83]. The input plane included both the object to be encrypted and the key, without the necessity of a lens or other optical components between input and output planes. The performance of the system was analyzed as a function of the distances from the object and key, respectively, to the CMOS camera. The system was validated with both simulations and experiments [83].

In security applications of optical correlators, the Gyrator transform was also used. A nonlinear image encryption system was proposed, based on a fully phase nonzero-order JTC in the Gyrator domain that does not require beam splitting as in other JTC proposals [84]. Two nonlinear operations modify the joint Gyrator power distribution (JGPD), enhancing both the security of the system against attacks and the quality of the decrypted image. The scheme uses three security keys: two RPMs and the angle of rotation of the GT. The setup was validated by numerical simulations [84]. The same research group introduced new operators (generalized shift, correlation, and convolution) based on the Gyrator transform and presented the obtained results for a nonlinear JTC used for optical encryption. The system was validated by numerical simulations [85]. As for the JFTC [78], the influence of noise and occlusion was analyzed also for the JTC based on the Gyrator transform [86]. The results showed a slightly better quality of the decrypted image when affected by multiplicative noise as compared to the additive one. Similar to the JFTC, the quality of the image is still acceptably preserved when the occlusion covers up to a quarter of the image area [86].

The Fractional Fourier domain has also been used for optical correlators in cryptosystems. Vilardy et al. [87] proposed an extension of the optical JTC cryptosystem to the fractional Fourier domain. Two different approaches were presented and, by combining them, the decrypted image quality and the security of the system were shown to increase. A protocol of optical encryption was proposed to remove speckle noise, allow multiple users, and increase the security of the system, by using a data container, multiplexing techniques and the JFrTC (Joint fractional Fourier transform correlator). The fractional order of the transform was used as an additional security parameter. The system was validated by experimental results [36]. The JFrTC was also used in conjunction with the VSS (visual secret sharing) technique in a novel method for data hiding, with an enhanced degree of security [88]. Using the VSS technique, two sharing data were generated, starting from both the cover and hidden images, and then the sharing data were further encrypted by applying a JFrTC. The fractional order of the transform (taken as the distance between the lens and the SLM or CCD) was also used as security parameter. The method was validated by numerical simulations (Matlab R2011b) [88].

More recently, the JFrTC was implemented for the first time in an architecture of a focus-tunable optical cryptosystem, with the aid of an electrically focus-tunable lens (EFTL) [89]. The fractional order of the fractional Fourier transform was also used here as a security parameter and the EFTL enabled its faster control as compared to mechanical solutions. Thus, the proposed cryptosystem is more stable and with reduced requirements for alignment as compared to previous ones. The processes of encryption and decryption were achieved with a wide range of values for the focal length of the EFTL and, based on these variations, a multi-user protocol was proposed. The system was validated by both simulations and experimental results [89]. A JTC encryption system, based on the Collins diffraction transform (CDT), was recently proposed [31]. As compared to a similar system that uses the Fourier transform instead of the Collins one, a threefold increase was observed for the maximum number of new security keys that can be introduced, while maintaining the property of invariance to translations both during decryption and retrieval of the original image, obtained with a very good quality. The architecture was validated by means of simulations [31]. Table 2 presents a comparison of the parameters chosen for the components of the optical setup in various proposals of JTC cryptosystem architectures based on other domains than the Fourier one.

As compared to the JTC cryptosystem, I remark that there are much fewer experimental implementations, the feasibility of the proposals being confirmed also by means of numerical simulations. The most frequently used is the JFTC correlator. The choices for the laser, SLM and camera are in most cases the same as the most commonly ones in JTC cryptosystems. As concerns the transformative lens, the JFTC has the advantages of not requiring one, thus simplifying the system.

#### 3.1.3. JTC Correlators for Image Recognition Applications

Although recently the most used application for the JTC correlator has been in optical cryptosystems, in the latest years there have been numerous proposals of the JTC also for image recognition purposes. The classical JTC correlator architecture was employed by several research groups in novel applications. Layton et al. [3] proposed, for the first time, the use of a JTC correlator for detecting the distance to an object. The results confirmed that the information about distance can be recovered from the output correlation peak of a JTC. Zhang et al. [90] elaborated a method for estimating the point spread function (PSF) based on a JTC and eliminated the problem of image quality deterioration due to missile vibration in a star map, obtaining an improvement of the PSNR (peak signal-to-noise ratio) of 26.50% as compared to a fuzzy star image.

JTC correlators based on Mach–Zehnder interferometry techniques (MZJTC correlators) have also been proposed recently in applications of object recognition. Such a correlator can perform the recognition of targets in the YIQ (luminance in-phase quadrature) color space, with invariance to distortions. Thus, an algorithm of optimizing the average cross-correlation was proposed [22]. For each color channel, the reference function was trained with different images, with a rotation range of 28 degrees, from −14° to 14°, with a step of 2 degrees. The proposed method was validated by numerical simulations, that showed a significant improvement of the PCE (peak-to-correlation energy) in the YIQ color space as compared to the conventional one based on RGB (red-green-blue) [22]. Earlier research by the same group had proposed a simulated annealing algorithm for pattern recognition using the MZJTC correlator in the YIQ color space and had shown, by numerical simulations, a significant improvement of the PSR (peak-to-sidelobe ratio) in the YIQ color space as compared to the RGB one [91].

Applications of image recognition have also used recently the JTC Cambridge correlator architecture. One of them is a system capable of recognizing microchip patterns (used in ID, SIM, and credit cards) according to specified criteria, based on the Cambridge correlator. By using the correlator, a comparison is performed between the input scenes, processed by software created in C# to extract the patterns from the static image, and the reference microchip patterns, stored in a database [42]. The same research group employed the Cambridge correlator in an application of an industrial system for video surveillance, to compare the dimensions of pavements, captured by a video camera and pre-processed with the Fourier Optics Experimenter software, with the ones of reference images stored in a database, benefitting from the possibility to process data at a high speed, in real time. A statistical approach was performed based on 100 measurements for each of the twenty pavements with different dimensions used in the experiment and a threshold of 92% was chosen for accepting or rejecting each pavement for the production process [43]. The Cambridge correlator was also used in an application of DNA and iris recognition, based on the same principle of comparing the input images with the reference ones from a database [44]. The same group used the Cambridge correlator in a video surveillance system, designed for detecting abandoned or lost baggage. The system is capable of detecting persons and their baggage and tracking their movements, by keeping track of their spatial coordinates. An experiment was performed, with four sets of inputs, each with two persons and three types of baggage [92].

Another application of the Cambridge correlator involved the development of a system for automatic detection and classification of traffic signs, together with the determination of their precise location and condition. The cross correlation between two pictures gives their similarity rate, resulting from the correlation peaks at the output of the optical correlator [93]. Also based on the Cambridge correlator, vertical traffic signs were successfully detected by comparing them with reference ones from a database. Three different detection methods were employed, based on color, shape, and a combination between them [94]. Another type of JTC correlator used in image applications is the PR-TBCJTC (photorefractive two-beam coupling JTC). Earlier studies with this correlator confirmed its efficiency in detecting simple images. Nehmetallah et al. [41] proposed an extension to the recognition of more complicated images and demonstrated, for the first time, a simultaneous enhancement of all the analyzed parameters by several orders of magnitude as compared to the traditional JTC architecture. Both simulations and experiments validated the proposed approach. The FJTC (fringe-adjusted JTC) correlator was also employed in image recognition applications. Moniruzzaman and Alam [38] proposed a technique of correlation with a FJTC based on PSK (four-phase shift keying) for face recognition. Experimental results, with different image datasets (Yale, Extended Yale B), in different conditions (variations of the incident light, distortions of face expression in 3D), showed a significant improvement of the performance parameters (PCE: peak-to-correlation energy, PCR: peak-to-clutter ratio, CPI: correlation peak intensity) as compared to classical techniques based on the JTC. The nonlinear JTC based on the SHG (second-harmonic generation) was also employed recently for applications of face and QR code recognition. Ref. [95] proposed such an application using as light source an IR (infrared) laser (1064 nm), with a KTP (potassium titanyl phosphate) crystal where SHG is performed, with the second-harmonic wave (532 nm), in visible light, used to obtain the output correlation signal. Other possible applications of the system, envisioned by the authors, are in security verification, optical computing, remote sensing, IR pattern recognition and remote sensing. Based on the nonlinear JTC (NL-JTC) technique, a scaled composite JTC was proposed for detecting the heads of swimmers [96]. The scaled composite JTC included in the input plane both the target and reference images (from a database) to be compared. The reliability of the proposed system was validated by tests performed on real sequences of video from the French swimming championships. The work represented an improvement on previous results of the same research group [97,98] where optimized versions of a swimmer tracking system were introduced.

Table 3 presents a comparison of the parameters chosen for the components of the optical setup in various proposals of JTC correlators used in image recognition applications.

As compared to the JTC cryptosystem (Table 1) and the other variations of the JTC for security applications (Table 2), I remark that there are much fewer experimental implementations to confirm the validity of the proposals. On the other hand, concerning the laser used in the setup, I have here for the first time a laser that operates outside the visible range (in IR), in the SHG-based JTC, where the wavelength is, however, reduced at half (532 nm) when reaching the output correlation plane.

#### 3.1.4. JTC Correlators for Other Applications

**Figure 9:**
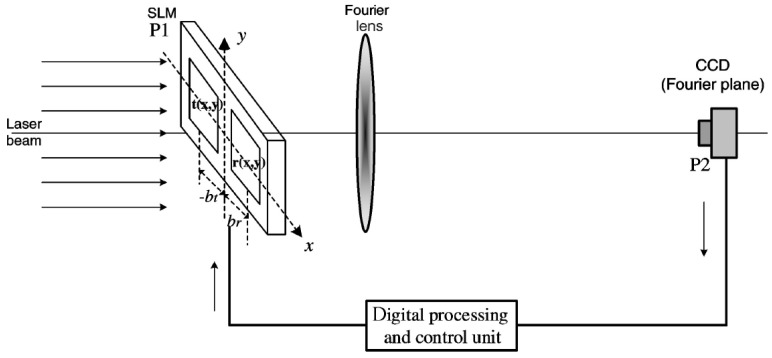
Proposed setup of a compact JTC architecture for spatial applications [101].

JTC correlators have benefited recently from renewed research interest also in domains such as medicine, space, or artificial intelligence (AI). In the medical domain, where the need for precision is high, JTC correlators can prove helpful. Such a system can place the image of the patient in the object plane, where it is illuminated by the incident light from a source. Secondly, the Fourier transform is performed by a biconvex lens placed between the object and Fourier planes. An LCD system is connected to a computer, placed under an angle of 45 degrees before another biconvex lens. At the output, a decision can be made concerning the health condition of the analyzed patient [99]. In another application using a CBJTC (compression-based JTC), Kaewphaluk et al. [40] demonstrated experimentally its robustness to noise in applications of retinal recognition in tele-ophthalmology. JTC correlators were also used for DNA sequence recognition [37]. DNA sequences were converted into 2D images. A combination of the target and reference DNA sequences was presented at the input of the JTC, as a joint input image. The similarity degree between the two images and the alignment between sequences were determined by the JTC. The method was validated by numerical simulations. In the spatial domain, several applications of the JTC have been proposed lately. Lis et al. [100] included an optical correlator in their system developed for a telescope mission, where hard X-rays are monitored. Fan [101] designed a compact JTC (having only one SLM, one Fourier lens, a CCD camera and a unit for digital processing and control, as in Figure 9) to measure the shifts, at a subpixel level, between two adjoining images, with the purpose of enhancing the quality of images taken by spatial cameras. The experimental results showed a very precise measurement of these shifts: an error of less than 0.1 subpixel for a shift under 1 pixel. Fan [102] further studied the influence of noise on the precision of measuring, with the JTC correlator, image shifts. Thus, both simulations and experiments were performed when the input images included added pattern noise and white background Gaussian noise. The results showed an error of less than 0.12 pel even in the conditions of an SNR (signal-to-noise ratio) of 1 dB for the input images. Dalei et al. [103] introduced a novel method for measuring the image motion of space cameras used in satellites, also employing a JTC optical correlator. The principle was based on subpixel correlation, together with global and adaptive thresholding and segmentation, to precisely identify the correlation peaks obtained at the output of the JTC. The method was confirmed by both simulations and experiments, with an error in measuring the image motion of less than 0.25 pixels.

In the domain of artificial intelligence (AI), recently a novel application of the nonlinear JTC correlator was proposed by George et al. [46]. Since the traditional JTC architecture is iterative and thus impractical for AI systems, the paper introduced a processor capable of executing the convolution in the optical domain, with a very low latency (close to zero), benefitting from the nonlinearity of the correlator. The method showed a reduction of the complexity scaling in processing 2D data and presented the ability to process in parallel millions of channels, in a non-iterative way, making it ideal for tasks of ML (machine-learning) involving a large matrix. These characteristics make such an ML accelerator attractive for future applications in cloud AI and network-edge systems. Table 4 presents a comparison of the parameters chosen for the components of the optical setup in various proposals of JTC correlators used in medical and spatial applications (for the AI application presented here, no experimental data were available).

From Table 4 one can observe that the laser type is the same as the most used one in security applications (Table 1): based on helium and neon and operating at a wavelength of 632.8 nm. As concerns the SLM and CCD, in some of these applications special types are employed, different than the ones used in the proposals presented in the first three tables. Finally, one also remarks that, as before, only some of the papers include, besides simulations, an experimental part, with a real setup of optical correlator to validate the results.

### 3.2. VLC Optical Correlator Applications

The second major class of optical correlators—the VLC (VanderLugt) ones—have also been represented lately in research work, in various applications of image recognition, the most frequent ones being the ones where the images to be recognized are human faces. In this section, I present several systems based on the VLC directed towards this purpose.

#### 3.2.1. VLC Optical Correlators for Face Recognition Applications

Face recognition is important in various situations. Some of the proposed applications concentrate on the recognition of the entire face, whereas others detect only a part, mainly the eyes. One such application was proposed by Ouabida et al. [104] to detect the state of drowsiness that might prove dangerous for fatigued drivers, as a cause of accidents. The suggested non-invasive method first detected the eyes, then estimated their state (opened or closed). Simulations were performed, using videos with real drivers, to detect drowsiness. The eye center was detected for the first time with the use of the VLC correlator in a numerical simulation. Moreover, correlation filters were designed and adjusted for each eye state with the purpose of eye recognition even in conditions of clutter and noise. The purpose was to find the best filter that offers a maximum value for the PSNR (peak signal-to-noise ratio). The eye state was estimated in several situations: different conditions of the illumination, different positions of the head, with and without eyeglasses. The VLC system offered the amplitude of the correlation peak as a criterion to detect the state of drowsiness of drivers. The proposed method proved an improved detection as compared to classical eye-tracking methods. The same research group [105] also proposed an algorithm for the segmentation and tracking of iris and pupil areas, with the method named OCAC (optical correlation-based active contours), where the correlator is a VLC one. The algorithm showed, by means of numerical simulations, an improved detection precision and less computational time as compared to other six algorithms proposed in literature for iris segmentation.

Various face recognition applications have also been proposed employing the VLC correlator. Ghorbel et al. [51] combined the VLC correlator and the GOM (Gabor ordinal measures) in a hybrid approach devised for face recognition and evaluated the system using two databases: FERET and YaleB extended. Both the computational time and the accuracy of recognition were improved as compared to systems using only GOM, achieving a recognition rate of 99.24% with the FERET database and 96.21% with YaleB extended. Kortli et al. [9] used the VLC correlator in a novel system, developed on a Zynq SoC (System-on-Chip) platform (designed by Xilinx), with the purpose of detection and recognition of faces with various poses, from −30° to 30°. The Viola–Jones detector was employed for locating and detecting faces. From the experimental results, 4.4 times increase in speed was observed as compared to the classical VLC (in its hardware implementation), and 39.22 times increase as compared to the classical VLC software implementation. Saumard et al. [6] introduced a novel method to improve the accuracy of the face recognition decisions based on the result in the VLC output correlation plane, by introducing a nonparametric model for this plane, by means of a kernel smoothing classification of the images in the plane, that considered the distribution and energy shape in this plane. The tests employing a classical POF (phase-only filter) confirmed the reliability of the method, with a low rate of false alarms (a mean square error MSE under 1%) and accurate detection. Jridi et al. [1] proposed, for face recognition applications, a more compact VLC architecture with only one lens instead of two. To achieve this, the Fourier plane was adjusted such that the correlation decision can be made in this plane. As advantages of the proposed setup, the authors mention: the simplification of the setup; the elimination of constraints due to a second lens; the improved adaptability due to the possibility of performing digitally the multiplication with the correlation filter; the elimination of the need to perform the IFT (inverse Fourier transform); a reduction by 100 times of the number of arithmetic operators and a better accuracy as compared to the classical approach. Moreover, the classical PCE (peak-to-correlation energy) decision metric was replaced for the first time with a comparison between the filtered spectra of the target image and reference image. Napoléon and Alfalou [52] created a new algorithm for face recognition, able to accurately reconstruct faces in various poses (different rotations of the head). A 3D mesh was built for the human face using a single photo. A VLC correlator was used for the identification, together with the techniques of DoG (difference of Gaussian filtering) and LBP (local binary pattern), and achieved a result of 88.76% accurate detections, as compared to only 44.97% with a classical 2D VLC counterpart. Bouzidi et al. [2] added a joint phase of pre-processing in the input plane of the VLC correlator, to enhance its robustness in applications of face recognition. Numerous tests were performed, using the PHPID (Pointing Head Pose Image Database) and several rotations of the faces, both horizontally and vertically, and validated the increased performance of this proposal (a more intense correlation peak at the output when the target and reference images match; a high background noise when they do not; an increase of the number of correct decisions and a reduction of the false ones), as compared to the classical VLC. Ghorbel et al. [49] employed four different methods for face recognition: two global ones: VLC and fractional eigenfaces, and two local ones: GOM (Gabor ordinal measures) and uLBP (uniform local binary pattern). Using the FERET database, all methods were tested for robustness to three different factors: variations of the illumination, different facial expressions and aging. The best results for the recognition rate were obtained for the GOM method, for all four sets of probes taken from the FERET database, as shown in Table 5.

Table 6 summarizes the obtained results for the aforementioned techniques of face recognition based on the VLC. For comparison purposes, the results from a few earlier papers were also shown. I also included an initial version of this table in one of my recent papers [5], that I now arranged and extended with new information. The legend of the table is as follows: techniques: VLC, VanderLugt correlator; GOM, Gabor ordinal measures; LBP, local binary pattern; DoG, Difference of Gaussian filtering; ZNCC: zero-mean normalized cross-correlation; databases: FERET, Face Recognition Technology; PHPID, Pointing Head Pose Image Database; matched filter or criterion used for image matching: PCE, peak-to-correlation energy; ASPOF, asymmetric segmented phase-only filter; POF, phase-only filter.

For the VLC correlator, as compared to the applications for the JTC, one can observe that the researchers preferred numerical implementations instead of developing an experimental setup, which has an increased complexity. Therefore, I could not offer in this chapter a summative and comparative table of the components used in different proposals, as in the previous sections.

#### 3.2.2. Different Types of VLC Optical Correlators for Image Recognition in Various Applications

As in the case of the JTC correlator, where several different alternative architectures have been proposed to replace the traditional one, to improve its performance, also different changes and enhancements have been introduced lately to the classical VLC correlator architecture, with the same purpose, but with a narrower range of setup alternatives as compared to the numerous JTC variations. Han et al. [50] studied a VLC correlator to enhance the performance of polarization imaging in underwater conditions, mainly when there is no detectable difference between the brightest and darkest moments during the rotation of the polarizer. The proposed solution aids to determine the optimal pair of orthogonally polarized images and can accurately locate the position of the optimal images and recover them with very good image quality and contrast. Goncharov et al. [110] analyzed, by means of numerical simulations, the implementation characteristics of a VLC correlator based on an amplitude liquid crystal (LC) SLM (HOLOEYE LC 2002), employed for displaying the input images in one considered case, and the holograms of the correlation filter in other two cases, with a MACH (maximum average correlation height) and a MINACE (minimum noise and correlation energy) filter, respectively. The authors also proposed a method to optimize the filters, to avoid errors in recognition due to additional phase modulation of light present in amplitude LC SLMs. Khonin [111] modelled a VLC correlator based on the Hilbert transform, applied for visualizing phase objects. The optical implementation of the Hilbert transform can be obtained by using a confocal system, with two lenses and, between them, a spatial optical filter. The purpose of the first lens is to place in its focal plane the input image. The output image is recorded by taking advantage of the second lens. The partial Hilbert transform can be modelled with Equations (3) and (4).

(3) $$H_{P0}\left(\xi \right)=isgn\left(\xi \right)$$

(4) $$H_{P0}\left(\xi ,\eta \right)=isgn\left(\eta \xi \right)$$

The radial Hilbert transform can be recorded, most readily, using polar coordinates, as in Equation (5).

(5) $$H_{V}\left(r,\Phi \right)=exp\left(i\Phi \right)$$

The parameters in these expressions can be obtained as: ξ=rcos(Φ) and η=rsin(Φ), respectively. The Hilbert radial transform can be implemented using a spiral phase plate (SSP) and can show distinctly both the phase contrast and the contours of an object [111]. In [111], the Hilbert transform was used in an optical correlator and a comparison was made between the effects of Zernike and vortex filters, where the latter obtain, using the Hilbert transform, information not only about the phase, as in a Zernike filter, but also about the amplitude of the wavefront. Thus, additional data can be gathered about the input image, with the use of the directional Hilbert transform to select the contours of the image in a specified direction. In one of my recent papers [112], I studied the implementation of the Hilbert transform in an optical correlator and its advantages. Based on the classical Hilbert correlator principle, I assessed the performance of an optical correlator, either JTC or VLC, based on the Hilbert transform in applications designed for image recognition. By means of MATLAB simulations, such an optical correlator was analyzed, confirming its efficiency and reliability in optical image recognition applications. Yang et al. [7] proposed, as a solution to the problem of target recognition in conditions of large angle rotation distortions, to replace the filter with a novel one based on a neural network (which was named neural network rotation recognition filter, NNRRF). As compared to a traditional OTSDF (optimized trade-off synthetic discriminant function) filter, an up to 1402.4% increase of the correlation peak index was observed. As compared to the NNCRF (neural network correlation recognition filter), proposed more recently by [113] (which in turn had obtained a 306.7% increase as compared to the OTSDF filter), the NNRRF achieved better performance in terms of the recognition ability invariant to distortions. The proposed filter was used in a planar integrated 2f optical correlator and was validated by experiments in a real setup. Xu et al. [48] presented an optical correlator with a 2f planar integrated structure, using an OTSDF filter, to enhance the integration of the setup and its ability to recognize patterns invariant to distortions. Instead of physical lenses, two specific digital microlenses were used (as in [7]). Additionally, a CCD and two SLMs were integrated on a substrate. To simplify the structure, both the microlenses and the data for the image and filter were loaded on the SLMs. A theoretical analysis was performed to obtain the main parameters of the device and its design characteristics. A volume of only 63.1 cm^3^ was obtained for the system, which is about half as compared to the classic 4f planar integrated correlator. Both theory and experiments confirmed the invariance to distortions of the compact integrated setup proposed for the optical correlator, with promising future applications in AI and machine vision. Essadike et al. [114] used for the first time a VLC correlator in a medical application with a novel automatic process of brain tumor segmentation. The tissue regions with abnormalities were automatically detected using a numerical simulation of the VLC, with a tumor filter adapted to all types of brain tumors, with a special focus on the most aggressive one, glioblastoma. The initial contour from the affected tissue were estimated from the correlation between this filter and the images obtained with MRI (magnetic resonance imaging). The results validated the accuracy of tumor detection and the improved performance as compared to other methods based on active contour. Xu et al. [47] introduced an integrated zigzag VLC (IZVLC) with an SLM to reduce the influence on the performance of the components’ misalignment. The SLM was used as a programmable filter. The Fourier transform functions were obtained with two refractive lenses. In this way, the volume of the VLC was miniaturized, and better performance was achieved in terms of recognition of patterns as compared to the classical VLC. A simulation model was created, and it was shown that the sensitivity of the setup is higher for the transversal misalignment as compared to the longitudinal one.

Table 7 presents a comparison of the parameters chosen for the components of the optical setup in various types of VLC correlators for image recognition in different applications. As mentioned above, very few experimental implementations of the VLC correlator have been proposed recently in literature research.

From Table 7 one can observe that the laser type is the same as the most used one in JTC correlator applications: the laser based on helium and neon, operating at a wavelength of 632.8 nm. One also remarks that, as compared to the JTC applications, the ones based on VLC proposed recently include in the most part theoretical analyses and numerical simulations, with only two recent papers including an experimental setup to validate the results.

### 3.3. Other Types of Optical Correlators—Implementation and Applications

Besides the most used two types of optical correlators (JTC and VLC), in the latest years also several applications (less numerous than for the JTC and VLC) have been proposed and implemented for other types of optical correlators. In this section, I will analyze applications involving the optical correlator in incoherent light, the holographic correlator, the HOC (hybrid opto-electronic) correlator and the WOC (window-based optical correlator), respectively.

#### 3.3.1. Optical Correlator in Incoherent Light for Object Recognition

**Figure 10:**
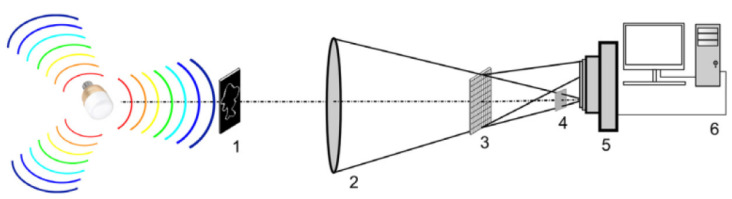
Setup of the incoherent light optical correlator proposed by Cheremkhin et al. [56]. 1: object to be recognized; 2: lens; 3 and 4: holographic and neutral filters, respectively; 5: camera; 6: PC.

A few different architectures of optical correlators in incoherent light have been proposed lately. Gnatovskiy et al. [55] employed a diffractive optical correlator in incoherent light for the detection of small displacements of objects. To accurately determine these displacements, they developed a method, based on the mutual spatial shift, in the transverse direction, between two diffraction gratings contained in the correlator setup. Cheremkhin et al. [56] proposed a dispersive optical correlator in incoherent light for object recognition. As radiation sources, LEDs (light emitting diodes) were used, instead of lasers as in the JTC and VLC correlators, because they emit incoherent light and have a broad spectrum. The authors developed an experimental setup that I present in Figure 10.

The setup includes a single lens, placed between the input plane (where the object is placed) and frequency plane (where the synthesized hologram is placed). The purpose of the neutral filter is for zero diffraction order light attenuation. The camera registered the output signals and the computer processed them. The results showed a correct identification of each object. Molodtsov and Rodin [53] implemented an optical correlator in incoherent light based on a special type of SLM, the DMD (digital micromirror device) spatial light modulator. Experiments were performed, that showed the necessity of determining the position and dimensions of the flattest area of the DMD matrix and to use only this area for hologram outputs, since if the entire surface, which is not perfectly flat, is used, the resolution of the system deteriorates. The efficiency of object recognition with this correlator in incoherent light was also validated by the experiments.

#### 3.3.2. Holographic Correlator Implementations

Several implementations were also proposed lately for the holographic correlator. Inoue et al. [115] experimentally developed for the first time a holographic correlator in an application of computational ghost imaging (CGI), of high speed, for applications involving moving objects. The RMSE (real mean square error) of the reconstructed image was evaluated using the correlator. A 133.7 fps imaging frame rate was estimated in the case of using 1000 random binary patterns for the acquisition of the reconstructed image. Ikeda et al. [4] achieved an increase of the stability of an optical correlator using a coaxial holographic memory, by introducing a new simple disc structure. The experimental results validated the detection efficiency of the proposed setup. Hoshizawa et al. [57] obtained an increase of the correlation speed in a holographic optical correlator, by introducing a method of data interleaving, that considered the rate of correlation with the image data last recorded to regroup the image data order of recording. Both experiments and simulations confirmed an increase by 1.33 times of the correlation speed as compared to the best previous architectures. Ikeda and Watanabe [116] developed an optical correlator using a coaxial holographic memory, obtaining an ultrahigh speed of computation of 142.9 Gbps. A comparison was established with a conventional system that used a central processing unit (CPU, 2.40 GHz x4) with a random access memory (RAM) of 16 GB, and showed a significant increase in the computation speed, especially for large-scale data.

Another version of the holographic optical correlator is a 3D one (with two spatial dimensions, together with the temporal dimension), i.e., a spatio-temporal correlator (STC). This correlator is capable of the recognition of the presence of a short clip inside a longer video. It presents both analogical and digital versions. In one of the analogical approaches [117], a 3D STC was proposed, using a combination between holographic correlation and temporal pattern recognition, the latter one based on photon echo. Thus, an AER (automatic event recognition) system was obtained, that achieved spatio-temporal invariance to shift, with the further possibility to also achieve spatial invariance to both rotation and scale, by using the polar Mellin transform. The STC is capable of fast recognition of events, such as a short clip from a long video and of determining its temporal location. The system was validated by numerical simulations. The authors also presented a method for obtaining a practical version of the device [117]. The same group developed an analytical method, using the STFT (spatio-temporal Fourier transform), and obtained an improvement with many orders of magnitude of the response speed of an AER system based on the STC, as compared to numerical methods based on the Schrödinger equation [118]. Alternatively, digital approaches have also been proposed. Video sequences can be represented as trajectories in a space with a high number of dimensions, by using scaled luminance fields. The search can be localized by using an indexing scheme, based on a kd-tree method, and can achieve a retrieval of the video content with high precision and at a high speed [119]. An index structure based on the mrkd-tree to search short clips in a larger video database can also be efficient and robust [120].

#### 3.3.3. Hybrid Opto-Electronic (HOC) Correlator Applications for Target Recognition

Various architectures of hybrid opto-electronic (HOC) correlators have also been proposed. Gamboa et al. [58] obtained target recognition with invariance to scale, shift and rotation using a HOC correlator. First, an opto-electronic combination of signal processing was employed to obtain the PMT (polar Mellin transformed) versions of two images. One of these PMT images was used as input and the other one as reference for the correlator. An experimental setup was developed, including a DPSS laser, a beam splitter, piezoelectric transducers, a Mach–Zehnder interferometer, photodetectors and a PID (Proportional-Integral-Differential) controller, and validated the efficiency and accuracy of recognition with the proposed method. To further improve the operational speed of the correlator, the same group [121,122] proposed the use, as optical storage database of high capacity, a holographic memory device (HMD) based on the polymer substrate PQ:PMMA (Phenanthrenequinone-doped poly(methyl methacrylate)), and experimentally validated the feasibility and efficiency of introducing, in the HOC correlator, the PQ:PMMA HMD. Monjur et al. [8] validated, by means of experiments, the functioning principle of a HOC correlator previously proposed by the same group. Sharp and lower correlation peaks, respectively, were observed in the matched and unmatched cases when the input and reference images were compared, with a 14 times higher amplitude of the peaks in the output signal for the matched case. Ortiz et al. [59] proposed another type of HOC correlator for microwave interferometry, as a part of an interferometer prototype for CMB (cosmic microwave background) applications. Experimental measurements validated the proposed setup. The prototype was further developed by the same group [123]. For frequency up-conversion, the Mach–Zehnder modulators were replaced by an electro-optical module. Signals were correlated and detected in the optical stage, at wavelengths in the NIR (near infrared) range, at 1550 nm. The method was validated by experiments.

#### 3.3.4. Window-Based Optical Correlator (WOC) for DNA Sequence Recognition

Another type of optical correlator, the WOC, has found an application in medicine for the recognition of DNA sequences. Mozafari et al. [16,17] proposed an algorithm based on two steps. In the first one, the DNA sequence was partitioned in several so-called windows. The second step evaluated the correlation with the reference sequence. The architecture included several optical correlators based on metamaterials, in parallel. The method was validated by means of simulations, proving an improved precision and speed, together with much less power consumption as compared to a classical method, based on the BLAST algorithm. The demonstrated increase of speed was with up to 60% as compared to the BLAST algorithm.

Summarizing the data presented in Section 3.3, Table 8 presents a comparison of the parameters chosen for the components of the optical setup in various proposals of optical correlators, other than the main two types (JTC and VLC), using available data in the papers and in the datasheets of the devices and components.

From Table 8 one can observe that, according to the various types of optical correlators, the characteristics can also vary significantly. Thus, among the optical correlators, the incoherent light optical correlator is the only one that can use an LED as light source, because LEDs emit non-monochromatic incoherent light; the holographic correlator has the specificity of using a holographic disc and two lasers (one for correlation and the other one for the recording on the holographic disc). Moreover, some of the correlator types (specifically, the HOC for microwave interferometry) also include in the setup optical fibers, that the JTC and VLC correlators do not have. One can also remark that, as opposed to the applications of JTC and VLC that used lasers with the most frequently chosen wavelength at 632.8 nm, the applications in Table 8 for other types of optical correlators use mainly lasers operating at lower wavelengths, typically 532 nm.

## 4. Challenges and Open Issues

Optical correlators of all types face various challenges. Some of them are characteristic for each specific major type of optical correlator (JTCs, VLCs, optical correlators in incoherent light). Others are specific for only some designs and architectures. Moreover, some general disadvantages of JTC or VLC have been overcome by specific proposals, whereas in other cases such perspectives are still envisioned as future developments. Some of the general challenges of optical correlators include difficulties in acquiring the perfect alignment of components in the setup; the cost of some of these components (such as the SLM); the redundancy of the transformations (from the spatial to the spectral domain, and from the spectral to the spatial one) [1]; the slow response of the components and their bulky characteristics. A faster response and smaller dimensions were achieved using components based on metamaterials [16,17]. The other disadvantages were alleviated in several proposals, but without a complete elimination. As concerns the JTC, several difficulties have been reported. The most significant ones are the noise that leads to a decrease in the quality of the decrypted image in a JTC cryptosystem, together with the vulnerability to various attacks, mainly due to the encryption linearity. The proposed systems that eliminate this linearity suffer, on the other hand, by an increased complexity and have a higher price [29]. Alternatives to the JTC cryptosystem have been proposed to increase the robustness against attacks and the quality of the decrypted image, such as the JFTC and the JTC variations in the Fresnel, Fractional Fourier, and Gyrator domains, respectively. The VLC, used mainly in image recognition applications, can also face challenges related to the necessity of improving the recognition rate, due to incorrect evaluation of the correlation peaks in terms of their height, location, and shape, or to the influence of scale or rotation on the correlation results [6]. Moreover, as compared to the JTC, the alignment requirements for the components of the setup are more severe and difficult to achieve; therefore, in numerous novel applications of optical correlators, the JTC was used, where the alignment of the components is less strict. The optical correlator in incoherent light, although robust, invariant to translations, and with less stringent requirements of alignment as compared to the VLC, still faces the challenge of a relative inability to discern correctly when there are only slight differences between the analyzed images [10].

Below I present the main challenges, open issues, and proposals of future enhancement for representative setups, techniques and architectures of optical correlators reviewed in this paper. The system proposed in [34], based on the JTC, for encryption and authentication of multiple users, applied on gray images, can be further extended to color images, that include more information. Moreover, it is also necessary to study the correlation results not only for images symmetrically positioned, as performed, but also when the images are randomly placed. The approach proposed in [71], based on the JTC, for the encryption in parallel of multi-channel images, could be implemented in at least two future applications: one in the management of authorization at multiple levels, where several authorized users could gain access to the information by using multi-level keys, and the other application in a video encryption system, where the proposed method could be used to encrypt more frames from the encryption video in only one ciphertext. The customized data container proposed in [18], for a JTC cryptosystem, could open new perspectives for applications of techniques for optical security systems of high performance, by overcoming limitations due to noise and to the maximum amount of information to be simultaneously processed. To further improve the concept, more studies are needed, that should also consider the image quality at the output of the JTC in relation to the characteristics at the input of the correlator. The JTC proposed in [95], based on the SHG, for the recognition of QR codes and human faces, has possible future applications in security checking and different implementations of optical processing of information, such as in IR remote sensing. The scheme is, however, limited by the possible range of values for the acceptance angle of the KTP crystal used in the experiment. This angle depends, in turn, on the length of the crystal and on the condition of phase matching. The JTC optical correlator developed in [3] for the first time for the determination of the distance to an object was envisioned by the authors for future use in space applications, irrespective of the static character of the background.

The JTC correlator proposed by George et al. [46], using nonlinear materials, might prove useful, besides the optical processing domain, in future applications such as edge computing, machine vision or in technologies for smart homes. As concerns the machine learning domain, this optical correlator could be able to bring about a revolution in fields such as recognition of speech, translation of foreign languages, analysis of images in medicine, autonomous vehicles, robots, and many others. From among the alternatives to the JTC correlator in other spectral domains, the JFTC proposed in [81], for encryption in parallel and retrieval in a hierarchical manner of multi-channel images, confirmed in the paper by means of a theoretical analysis and simulations, might be further developed, and explored experimentally in other systems for optical security with multi-channel images. The JFrTC correlator simulated in [88], using a VSS technique, might also be improved, as the authors proposed, for obtaining a higher precision in the reconstruction of the correlation result. Moreover, each JTC correlator (classical or in alternative setups) that was tested only by means of simulations might be also implemented in an experimental setup to validate the simulation results, perform additional tests, and include or apply in several novel directions. Concerning the limitations and future extensions of applications involving the VLC correlator, the system introduced by Napoléon and Alfalou [52] suffered from a lack of precision on the axis Oz of the 3D model, because, for building the 3D mesh, only one photo was employed. Thus, when the images are reconstructed, some depth information about the human face lacked accuracy, mainly for the nose. The authors proposed, as a future enhancement of the depth reconstruction, the addition of a profile view. Moreover, to be able to use a more substantial database, the addition of a nonlinear criterion-based decision step was also envisioned. The approach proposed in [51], based on the VLC in combination with the GOM technique, for recognition of faces, could be improved to achieve a lower computation time, while keeping the same high value of the recognition rate. As concerns the holographic optical correlator, Ikeda et al. [116] proposed future applications of their high-speed coaxial correlator in recognition and identification of image and video, respectively, in data processing techniques involving deep learning, and in various other applications that require an increased quantity of inner-product computations. Although the HOC correlator has recently achieved correlation with invariance to rotation, scale and position, future developments are aimed towards its complete automation for an operation in real-time. The speed is envisioned to be further improved, up to an ideal value of approximately 5 μs per correlation, using optoelectronic integrated circuits, customized for this type of correlator [121]. Another enhancement for the HOC correlator, proposed by Monjur et al. [8], was to include a new component, not yet developed, named the IGPU (Integrated Graphic Processing Unit) that enables an increase of the speed such that a correlation operation could be performed in only several microseconds, by avoiding the considerable slowing down due to serial communications between SLMs, FPGAs (field programmable gate arrays), cameras and other components of the correlator setup.

Another challenge is to step outside the laboratory, from research prototypes towards practical implementations and applications in the real world. One practical implementation of the JTC correlator, presented in our paper, is the Cambridge correlator, developed by Cambridge Correlators Ltd. (West Yorkshire, UK), that has found uses in medicine, in biometric recognition applications, in traffic signs recognition etc. [124].

Another optical correlator available commercially is OC-VGA3000, developed by INO (Institut National d’Optique, Sainte-Foy, Canada). It is a compact correlator, with an easy-to-use interface, 512 × 480 pixels processed area, and a 30 frames/second refresh rate [125].

A practical binary 256 × 256 optical correlator was also implemented, in 1998, by Boulder Nonlinear Systems, Inc. (Lafayette, CO, USA) It uses two SLMs for the input data, and a high-speed 256 × 256 Dalsa CCD camera at the output. Amplitude or phase modulation of light, provided by the SLMs, can be selected by rotating an analyzer at the output of the device [124].

For face recognition applications, a practical optical correlator was developed in 2007 by the Faculty of Sciences from Japan Women’s University, named FARCO (Fast Face-Recognition Optical Correlator). It is a hybrid opto-electronic correlator that uses a combination of optical correlation and a digital database, with a very good operating speed of 1000–5000 frames per second. An improved version, S-FARCO (Super Fast Face-Recognition Optical Correlator) was also developed, including a holographic optical memory [124].

In summary, optical correlators have the important advantages of being able to transfer data at a high speed, to analyze these data in an efficient manner, benefitting from the intrinsic property of parallel processing, the robustness to noise, a very good discrimination ability, power consumption requirements much lower than in the electronic counterparts, shift invariance etc.

On the other hand, optical correlators still suffer from disadvantages such as: the event of an incorrect decision is still possible when the output correlation peaks are analyzed in terms of location, shape, and height; the scale and rotation invariance have not yet been achieved; the alignment of the optical components in the setup is still challenging; the cost of some components (such as the SLM) is very high etc.

As concerns the current research and application status, there are some types of correlators that benefitted from a more extensive focus, such as the JTC applied in cryptography and the VLC used in face recognition applications. Some applications that were the leading ones some decades ago (such as military applications of missile detection; medical uses, e.g., for improvement of cancer tests; robotics; industry; antique scripts recognition; quality control; digital identification of fingerprints [10]) are much less researched today, as more efficient competitors of the optical correlators in these specific applications have taken their place. The same can be stated about other correlator types, such as the one in incoherent light, also relatively widely researched a few decades ago.

However, as development prospects, it could be predicted that the optical correlators would still be researched and novel applications developed, according to the new needs and new technologies where the unique properties of correlators can be taken use of. Nowadays the main application is for security purposes, in cryptosystems. As the security attacks are becoming more sophisticated and intrusive, it is possible that in the next decade this application of optical correlators will still be the leading one, together with other developments that will keep the pace with general advances in electronics and optics technologies.

## 5. Conclusions

In this paper, I presented a review of optical correlators. Firstly, I included general considerations regarding techniques of optical correlation, with an emphasis on the optical correlator as a device based on such techniques. Secondly, I clarified theoretical aspects concerning several types of optical correlators, detailing the different setup alternatives and sub-types, together with their main domains of applications. Thirdly, state-of-the-art implementations and proposals of all these optical correlator types were reviewed, with applications mainly in security and image recognition. Moreover, I emphasized the major strengths of the proposed techniques, methods and variations of optical correlator setups, and some of the factors that affect their performance, together with relevant directions for future enhancement. In one of my earlier papers [10] I had reviewed, for the first time, the domain of optical correlators with all their sub-types and the latest applications until that moment. Seventeen years after, to the best of our knowledge, our paper has remained the only extensive and comprehensive review of the domain. However, due to the passing of almost two decades, although the basics have remained unchanged, novel applications emerged, together with a shift from image recognition applications, dominant at that time, towards security applications. Therefore, an up-to-date survey of the field was absolutely necessary. Moreover, this approach is a much more detailed one, with the goal of helping any scientist who works in the domain, irrespectively if the employed correlator is the JTC, VLC or another, to easily find all the relevant information as a starting point for enhancing existing techniques and developing novel ones, to fully benefit from the advantages of these devices, highlighted in our paper.

## Figures and Tables

**Table 1:** Comparison between the most important parameters of the JTC setup in various proposed architectures.

Component	Type	Parameter	Value	References
Laser	He–Ne	Wavelength	632.8 nm	[26,33,63,67] [18,19,27,71,76]
	DPSS, Laserglow Technologies, output power 50 mW		542 nm	[74]
SLM	HOLOEYE PLUTO-VIS-014 (reflective phase-only SLM)	Resolution; pixel pitch	1920 × 1080; 8 μm	[26,63,67,76]
	HOLOEYE HEO-1080P phase-only SLM			[19]
	HOLOEYE LC 2002		800 × 600; 32 μm	[18,27]
	Phase-only SLM-R2		1280 × 1024; 12.3 μm	[71]
Camera	CCD (model N/A)	Resolution; pixel pitch	768 × 576; 8.3 μm	[26,33,63,67,71]
	CCD (PULNIX TM6703)		640 × 480; 9 μm	[27]
	CCD (pco.1600)		1600 × 1200; 7.4 μm	[19]
	CMOS EO-10012M		3480 × 2748; 1.67 μm	[74]
	CMOS EO-10012C			[18]
Lens	Fourier lens	Focal length	400 mm	[26,63,67,71]
			300 mm	[33]
			200 mm	[18,27,74]
			100 mm	[19]

**Table 2:** Comparison between the most important parameters of the JTC setup in various proposed architectures, other than the Fourier transform-based one.

Component	Type	Parameter	Value	References
Laser	He–Ne	Wavelength	632.8 nm	Fresnel: [30,82]
	DPSS		532 nm	Fresnel: [83]
	He–Ne (JDS UNIPHASE 1135)		632 nm	Fractional Fourier: [36,89]
SLM	HOLOEYE PLUTO-VIS-014 (reflective phase-only SLM)	Resolution; pixel pitch	1920 × 1080; 8 μm	Fresnel: [30,82]
	HOLOEYE 2002		800 × 600; 32 μm	Fresnel: [83]
				Fractional Fourier: [36,89]
Camera	CCD (model N/A)	Resolution; pixel pitch	768 × 576; 8.3 μm	Fresnel: [30]
			N/A	Fresnel: [82]
	CMOS (EO-10012M)		3840 × 2748; 1.67 μm	Fresnel: [83]
				Fractional Fourier: [36,89]
Lens	N/A	Focal length	200 mm	Fractional Fourier: [36]
	EFTL		variable	Fractional Fourier: [89]

**Table 3:** Comparison between the most important parameters of the JTC setup in various proposed architectures, in applications of image recognition.

Component	Type	Parameter	Value	Reference
Laser	LM635 laser diode with pigtail single-mode optical fiber	Wavelength	635 nm	Cambridge: [93]
	Cobolt Rumba laser		1064 nm	SHG-based JTC: [95]
SLM	SDE1024	Resolution; pixel pitch	1024 × 768; 9 μm	Cambridge: [93]
	Hamamatsu X10468-07		800 × 600; 20 μm;	SHG-based JTC: [95]
Camera	CMOS	Resolution; pixel pitch	656 × 494; N/A	Cambridge: [93]
	CCD (Thorlabs DCU224C)		1280 × 1024; 4.65 μm	SHG-based JTC: [95]
Lens	Fourier lens	Focal length	N/A	Cambridge: [93]
			100 mm	SHG-based JTC: [95]

**Table 4:** Comparison between the most important parameters of the JTC setup in various proposed architectures, in medical and spatial applications.

Component	Type	Parameter	Value	References
Laser	He–Ne	Wavelength	632.8 nm	Medical: [40]
				Spatial: [101,103]
SLM	Jenoptik EASLM (electrically addressed SLM)	Resolution; pixel pitch	832 × 624; 32 μm	Medical: [40]
	Forth Dimension Displays electrically addressed TFT-LCD XGA3		1024 × 768; 18 μm	Spatial: [101,103]
Camera	CCD (Pulnix TM2016-8)	Resolution; pixel pitch	1920 × 1080; 7.4 μm	Medical: [40]
	CCD (PULNIX TM6703)		768 × 512; 9 μm	Spatial: [101,103]
Lens	Fourier lens	Focal length	300 mm	Medical: [40]
			196.6 mm	Spatial: [101]
			N/A	Spatial: [103]

**Table 5:** Comparison between the recognition rate results obtained by Ghorbel et al. [49] with the VLC and other three methods for face recognition, with four different probe sets from the FERET database.

FERET Probe Set	VLC	Fractional Eigenfaces	GOM	uLBP
Fb	81.51%	84.2%	98.33%	93.39%
Fc	83.51%	25.25%	98.97%	98.45%
Dup1	73.82%	47.5%	92.52%	83.66%
Dup2	68.38%	9.82%	90.6%	82.91%

**Table 6:** Comparison between the most important characteristics of VLC techniques proposed for face recognition applications. I adapted the data from [9,20] and added new information. For an easier comparison of the results than in our early version [5], I now arranged the data in descending order of the percent of successful face recognition.

Technique	Database	Matching	Advantage	Limitation	Percent of Successful Face Recognition	Reference
VLC + GOM	FERET; Yale B Extended	PCE	Recognition rate	Computation time	99.24% 96.21%	[51]
LBP and VLC	YaleB; YaleB Extended	POF	Rotation + Translation	Illumination	98.40% 95.80%	[106]
VLC + Viola–Jones	PHPID	PCE	Increase in speed	Irrelevant information detected by the Viola–Jones detector	96% (software implementation); 94% (hardware/software co-design)	[9]
VLC correlator	PHPID	ASPOF	Robustness to noise	Too few reference images	92%	[107]
VLC + DoG + LBP	PHPID	POF	Robustness to illumination variations	Depth reconstruction	88.76%	[52]
VLC correlator	PHPID	POF	Reduced size of the filter	Aperture issues	88.27%	[108]
VLC correlator	PHPID	PCE	Processing time	Power	85% (for fixed images); 77% (for video sequences)	[109]
VLC + DoG	FERET	PCE	Robustness	Low rate of recognition	83.51%	[49]
VLC + ZNCC	PHPID	POF	Setup simplification	Low rate of recognition	75.38%	[1]

**Table 7:** Comparison between the most important parameters of the VLC setup in various proposed architectures, for image recognition in different applications.

Component	Type	Parameter	Value	Reference
Laser	He–Ne	Wavelength	632.8 nm	VLC with NNRRF filter: [7]
				VLC with OTSDF filter: [48]
SLM	LCOS reflective pure phase SLM (HOLOEYE-PLUTO)	Resolution; pixel pitch	1920 × 1080; 8 μm	VLC with NNRRF filter:[7]
				VLC with OTSDF filter: [48]
Camera	CCD	Resolution; pixel pitch	N/A	VLC with NNRRF filter: [7]
				VLC with OTSDF filter: [48]
Lens	Digital microlenses	Focal length	N/A	VLC with NNRRF filter: [7]
			170 mm; 340 mm	VLC with OTSDF filter: [48]

**Table 8:** Comparison between the most important parameters of the optical correlator setup in various proposed architectures, other than the JTC and VLC.

Component	Type	Parameter	Value	References
Laser	SFLS 1550S laser diode	Wavelength	542 nm	HOC: [59,123]
	N/A		532 nm	Incoherent light: [53]
	Q-switched laser (Spectra-Physics Navigator II J80-YHP70) for recording on the holographic disc; CW (continuous wave) laser (Showa Optronics J100GS-11) for correlation			Holographic: [57]
	Q-switched laser (Spectra-Physics Navigator II J80-YHP7) for recording on the holographic disc; CW (continuous wave) laser (Showa Optronics H6780-01) for correlation			Holographic: [115]
	Q-switched laser (for recording); CW laser (for correlation)			Holographic: [4]
	CW DPSS laser (Verdi V2)			HOC: [8,58]
LED	N/A		Broad spectrum	Incoherent light: [56]
SLM	DMD-SLM (DLP7000 0.7 XGA, Texas Instruments)	Resolution; pixel pitch	1024 × 768; 13.68 μm	Incoherent light: [53]
	Santec SLM-100		1440 × 1050; 10.4 μm	Holographic: [57,115]
Camera	CMOS (Canon EOS 400D)	Resolution; pixel pitch	3888 × 2592; N/A	Incoherent light: [56]
	CMOS (Thorlabs DCC1545M)		1280 × 1024; 5.2 μm	HOC: [8,58]
	Xenics Xeva NIR InGaAs camera		320 × 256; 30 μm	HOC: [59]
	Xenics Cheetah-640-CL		640 × 512; 20 μm	HOC: [123]
Lens	N/A	Focal length	210 mm	Incoherent light: [56]
	N/A		100 mm	HOC: [59,123]
	Edmund Optics 48137		4 mm	Holographic: [57]
Holographic disc	N/A	Thickness of the recording layer	0.5 mm	Holographic: [57]
Optical fiber	Optical fiber bundle (N/A)	Core diameter	N/A	HOC: [59,123]

## Data Availability

Not applicable.

## Abbreviations

The following abbreviations are used in this manuscript:

4-PSK WSJTC	four-phase shift-keying wavelet-filtered-based shifted-phase encoded joint transform correlator
AI	artificial intelligence
ASPOF	asymmetric segmented phase-only filter
B	baffle
BE	beam expander
BJTC	binary joint transform correlator
BPOF	binary phase-only filter
BS	beam splitter
BSL	Billet split lens
CBJTC	compression-based joint transform correlator
CCD	charge coupled device
CDD	Collins diffraction domain
CDT	Collins diffraction transform
CGI	computational ghost imaging
CL	collimated lens
CMB	cosmic microwave background
CMF	classical matched filter
COA	ciphertext-only attack
CPA	chosen-plaintext attack
CPI	correlation peak intensity
CPU	central processing unit
CS	collimation system
CSK	complex secret key
CTMF	complex ternary matched filter
CW	continuous wave
DMD	digital micromirror device
DNA	deoxyribonucleic acid
DoG	difference of Gaussian filtering
DPSS	diode pumped solid state
DRPE	double random phase encoding
EASLM	electrically addressed spatial light modulator
EFTL	electrically focus-tunable lens
ES	electronic subtractor
FARCO	Fast Face-Recognition Optical Correlator
FERET	Face Recognition Technology
FJTC	fringe-adjusted joint transform correlator
FL	Fourier lens
FOE	Fourier Optics Experimenter
FT	Fourier transform
FTL	Fourier transform lens
GD	Gyrator domain
GIE	ghost-imaging-based encryption
GOM	Gabor ordinal measures
GT	Gyrator transform
HMD	holographic memory device
HOC	hybrid opto-electronic correlator
HP	high-pass
HVD	holographic versatile disk
HWP	half wave plate
IFT	iterative Fourier transform
INO	Institut National d’Optique
IR	infrared
IZVLC	integrated zigzag VanderLugt correlator
JFPD	joint Fresnel power distribution
JFrTC	joint fractional Fourier transform correlator
JFTC	joint Fresnel transform correlator
JGPD	joint Gyrator power distribution
JPEG	Joint Photographic Experts Group
JPS	joint power spectrum
JTC	joint transform correlator
JTPS	joint transform power spectrum
KPA	known-plaintext attack
KTP	potassium titanyl phosphate
L	lens
LBP	local binary pattern
LC	liquid crystal
LED	light emitting diode
M	mirror
MACE	minimum average correlation energy
MACH	maximum average correlation height
MFC	matched filter correlator
MINACE	minimum noise and correlation energy
ML	machine-learning
MSE	mean-square error
MZJTC	Mach–Zehnder joint transform correlator
N0JTC	nonzero-order joint transform correlator
NIR	near infrared
NL-JTC	non-linear joint transform correlator
NNCRF	neural network correlation recognition filter
NNRRF	neural network rotation recognition filter
OCAC	optical correlation-based active contours
OTSDF	optimized trade-off synthetic discriminant function
PC	photon-counting
PCE	peak-to-correlation energy
PCR	peak-to-clutter ratio
PHPID	Pointing Head Pose Image Database
PID	Proportional-Integral-Differential
PMT	polar Mellin transformed
POF	phase-only filter
PQ:PMMA	phenanthrenequinone-doped poly(methyl methacrylate)
PR-TBCJTC	photorefractive two-beam coupling joint transform correlator
PSF	point spread function
PSNR	peak signal-to-noise ratio
PSR	peak-to-sidelobe ratio
PST	phase-shifting technique
QPF	quad phase filter
QR	Quick Response
QWP	quarter wave plate
RAM	random access memory
RGB	red-green-blue
RJTC	reference phase-encoded joint transform correlator
RLCSLM	reflective liquid crystal spatial light modulator
RLE	run-length encoding
RMSE	real mean square error
RPM	random phase mask
RSA	Rivest–Shamir–Adleman
S-FARCO	Super Fast Face-Recognition Optical Correlator
SHG	second-harmonic generation
SLM	spatial light modulator
SMF	single-mode fiber
SNR	signal-to-noise ratio
SoC	System-on-Chip
SPM	spiral phase mask
SSIM	structural similarity
SSPM	structured spiral phase mask
SVM	support vector machine
TSH	transverse second harmonic
TSHG	transverse second harmonic generation
uLBP	uniform local binary pattern
VLC	VanderLugt correlator
VSS	visual secret sharing
WOC	window-based optical correlator
ZNCC	zero-mean normalized cross-correlation
